# Adapting Physiology in Functional Human Islet Organogenesis

**DOI:** 10.3389/fcell.2022.854604

**Published:** 2022-04-26

**Authors:** Eiji Yoshihara

**Affiliations:** ^1^ Lundquist Institute for Biomedical Innovation at Harbor-UCLA Medical Center, Torrance, CA, United States; ^2^ David Geffen School of Medicine at University of California, Los Angeles, CA, United States

**Keywords:** human islet-like organoids, nuclear receptors, physiology, human pluripotent stem cells, diabetes

## Abstract

Generation of three-dimensional (3D)-structured functional human islets is expected to be an alternative cell source for cadaveric human islet transplantation for the treatment of insulin-dependent diabetes. Human pluripotent stem cells (hPSCs), such as human embryonic stem cells (hESCs) and human induced pluripotent stem cells (hiPSCs), offer infinite resources for newly synthesized human islets. Recent advancements in hPSCs technology have enabled direct differentiation to human islet-like clusters, which can sense glucose and secrete insulin, and those islet clusters can ameliorate diabetes when transplanted into rodents or non-human primates (NHPs). However, the generated hPSC-derived human islet-like clusters are functionally immature compared with primary human islets. There remains a challenge to establish a technology to create fully functional human islets *in vitro*, which are functionally and transcriptionally indistinguishable from cadaveric human islets. Understanding the complex differentiation and maturation pathway is necessary to generate fully functional human islets for a tremendous supply of high-quality human islets with less batch-to-batch difference for millions of patients. In this review, I summarized the current progress in the generation of 3D-structured human islets from pluripotent stem cells and discussed the importance of adapting physiology for *in vitro* functional human islet organogenesis and possible improvements with environmental cues.

## Introduction

**FIGURE 1:**
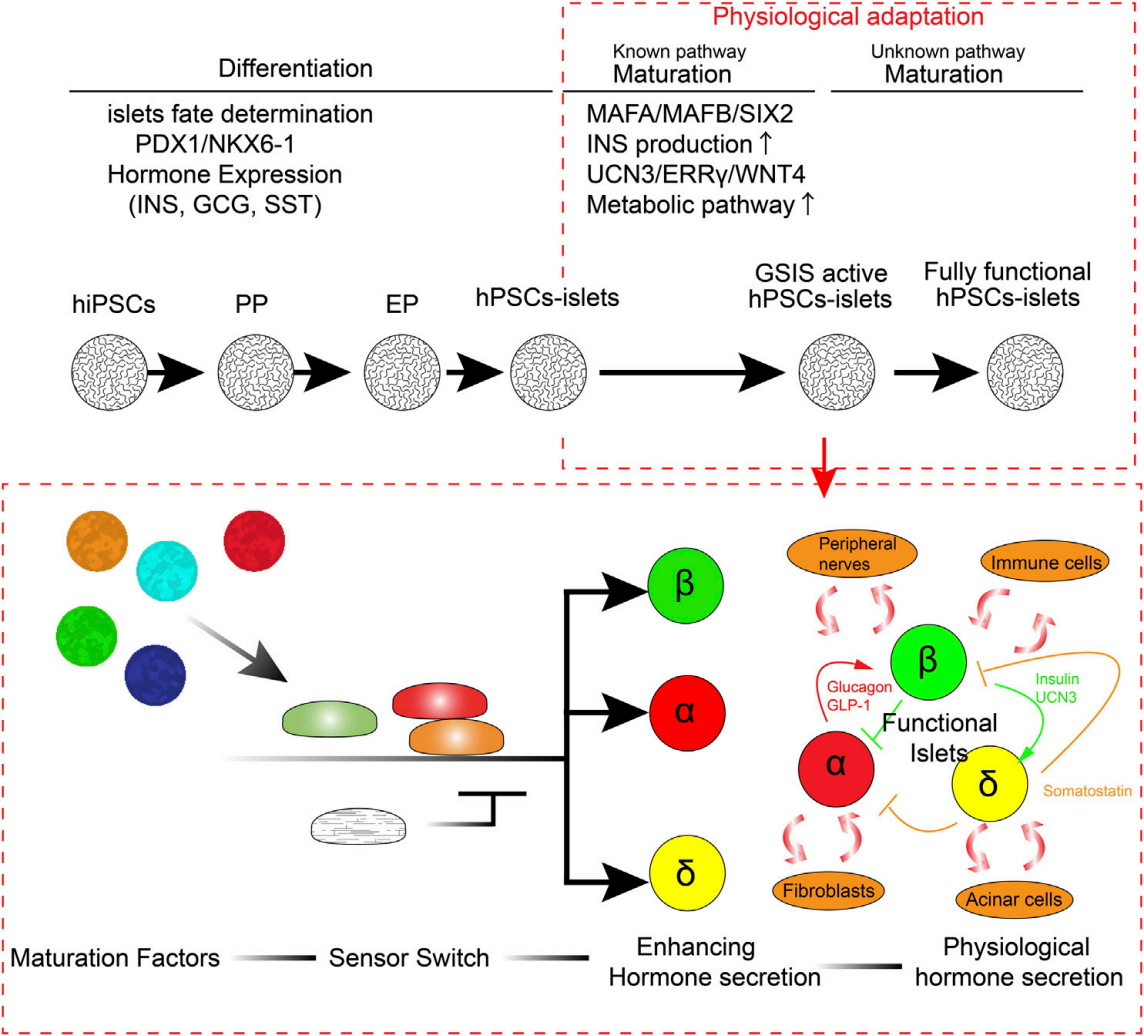
Stepwise physiological adaptation in functional human islet maturation.

Diabetes is a complicated chronic disease that affects more than 450 million people worldwide (Cho et al., 2018; Huang et al., 2018; Rogers et al., 2020; Rogers and Kim, 2020; Shah et al., 2020; Smalls et al., 2020; Tan et al., 2020). Microvasculature defects caused by diabetes lead to stroke, blindness, and kidney failure in some patients (Creager et al., 2003; Luscher et al., 2003; Beckman et al., 2013; Paneni et al., 2013; Cayabyab et al., 2021). Although the discovery of insulin a century ago made significant improvements in diabetic therapeutics, from lethal to treatable, even modern insulin therapy cannot be an alternative to endogenous functional insulin-producing β cells. Insulin treatment requires manual glucose monitoring and adjustment of insulin injection in both amount and timing every day, which also means the high maintenance and economic impact for the patients and expenditure on healthcare in all countries. Continuous, rapid fine-tuning of glucose or other nutrition-sensing insulin secretion is necessary for functional cure of diabetes. Cadaveric islet transplantation provides a functional cure for insulin-dependent type 1 diabetes (T1D) with a success rate of more than 90%, and its efficacy has been sustained for over 20 years in some cases (Lemos et al., 2021). The short supply and requirement of immunosuppressants, which increase the risk of adverse effects, limit the islet transplantation for the treatment of diabetes. Owing to their pluripotency and self-renewal function, human pluripotent stem cells (hPSCs), such as human induced pluripotent stem cells (hiPSCs) (Takahashi et al., 2007) and human embryonic stem cells (hESCs) (Thomson et al., 1998), offer an alternative cell resource for human cadaveric islets. Step-by-step treatment of small chemicals and recombinant proteins, which induce differentiation from hPSCs, was developed by integrating the knowledge of specific signaling pathways that contribute to the pancreatic lineage specification and islet differentiation. While recent advances in this approach have made it possible to reproducibly generate functional hPSC-derived insulin-producing β-like cells, which are capable of sensing glucose concentration and secreting insulin, there are several major limitations to our knowledge in generating fully functional human β cells or human islets. First, we have not yet clarified the pathway to induce each endocrine cell type separately. Although there are several reports on the production of more biasedly α cells rather than β cells from hPSCs (Rezania et al., 2011; Peterson et al., 2020), these established protocols do not generate a single-cell type but rather contain other cell types together with differentiation. Second, we have not completely understood the pathway of postnatal functional islet maturation, which typically occurs a few years after birth in humans. hPSC-derived insulin-producing cells are generated either by β-like cells (hPSC-derived β-like cells; monolayer or isolated insulin-producing cells) or human islet-like organoid clusters (hPSC-derived islets; α, β, γ-cells containing 3D-structured cell cluster). Therefore, the current protocol of generating fully functional hPSC-derived β-like cells or islets remains a challenge. Identifying such a pathway is necessary to improve the immaturity of generated islets and reduce the risk of batch-to-batch differences. In this review, I aimed to outline the current progress in generating functional human pancreatic islets from the perspective of 1) pancreatic lineage specification, 2) postnatal functional maturation, 3) differences between primary human islets and hPSC-derived islets, 4) missing factors, and 5) further adaptation of the environmental physiological responses (Figure 1). Additionally, I aimed to discuss future perspectives to improve the current protocol for generating functional human islets from hPSCs.

### Pancreatic Lineage Specification

Since the establishment of hESCs (Thomson et al., 1998), insulin-producing β cells have been identified in spontaneously differentiated cells from hESCs (Lumelsky et al., 2001; Assady et al., 2001). In addition with the efforts to identify the essential pathways for pancreatic lineage specification, efficient endodermal differentiation (D'Amour et al., 2005) and pancreatic progenitor (PP) differentiation (D'Amour et al., 2006) protocols have been developed. hESC- or hiPSC-derived PPs are capable to differentiate into mature functional β cells *in vivo* within a couple of months of transplantation (Kroon et al., 2008), suggesting that these protocols represent the right track for generating functional β cells *in vitro*. The key features of stage-specific differentiation are controlled by stage-specific transcriptional factors (TFs) and key identity genes (Tahbaz and Yoshihara, 2021). hPSCs undergo definitive endodermal (DE) differentiation by targeting the Activin A pathway and canonical Wnt (cWnt) pathway, Wnt3A. SRY-box transcription factor 17 (SOX17) and Forkhead box A2tbox2 (FOXA2) are prominent DE markers. Fibroblast growth factor 7 (FGF7/KGF) induces a primitive gut tube (PGT). Hepatocyte nuclear factor 1β (HNF1β) and hepatocyte nuclear factor-4α (HNF4α) are well-known PGT markers. Retinoic acid (RA) signaling activation, combined with sonic hedgehog (SSH) inhibition, bone morphogenetic protein (BMP) signal inhibition, and protein kinase C (PKC) activation, leads to PPs. PPs are pancreatic and duodenal homeobox 1 (PDX-1) and NK6 homeobox 1 (NKX6-1) double-positive cells, which can further generate mono-hormonal β cells, α cells (glucagon producing), and δ cells (somatostatin producing). RAs activation, SSH inhibition, BMP signal inhibition, transforming growth factor β (TGFβ) signal inhibition, and thyroid hormone signaling activation (THR) lead to endocrine progenitor (EP) generation. NEUROG3 (NGN3) is the key transcriptional factor of EP. It has been recently observed that targeting the cytoskeleton by inhibiting actin polymerization enhances NGN3 expression in the endocrine stage (Mamidi et al., 2018; Hogrebe et al., 2020). γ-secretase inhibition, BMP signal inhibition, TGFβ signal inhibition, and THR activation lead to the generation of endocrine hormone-producing cells (α, β, and γ cells). Pre-matured glucose-response β-like cells are generated (Pagliuca et al., 2014; Rezania et al., 2014; Russ et al., 2015). Although these previous studies established reproducible GSIS, impaired glucose-stimulated Ca^2+^ flux, reduced production of insulin, and many transcriptional differences have been observed in hPSC-derived β-like cells compared to those of primary β cells or islets (Veres et al., 2019; Augsornworawat et al., 2020; Yoshihara et al., 2020; Balboa et al., 2022). These results suggested that hPSC-derived β-like cells may remain a feature of the juvenile/neonatal stage of β cells (Hrvatin et al., 2014).

### Postnatal Functional Maturation

Increasing evidence suggests that multiple functional maturation pathways play an important role in the postnatal acquisition of robust GSIS function (Alvarez-Dominguez and Melton, 2021). Therefore, the current focus on improving the protocol for generating functional islets relies on the identification of the new pathways for functional maturation. Islet functional maturation is accelerated after birth to adapt to oxygen- and nutrition-rich environments. There are a number of transcriptional factors and key β-cell marker gene expressions such as *INSULIN* (*INS*), MAF bZIP transcription factor A (*MAFA*), SIX homeobox 2 (*SIX2*), glucose-6-phosphatase catalytic subunit 2 (*G6PC2*), and urocortin 3 (*UCN3*), which are lower in hPSC-derived β-like cells generated *in vitro* (Balboa et al., 2022). Chromatin remodeling and miRNA expression have been suggested to control the gene expression for functional β-cell maturation (Xie et al., 2013; Dhawan et al., 2015; Jacovetti et al., 2015). *MAFA* expression, regulated by T3 and other unknown signals, is important for *INS* and *G6PC2* transcription (Martin et al., 2008; Artner et al., 2010; Aguayo-Mazzucato et al., 2011; Hang and Stein, 2011). G6PC2 expression suppresses low-glucose concentration-stimulated insulin secretion, leading to amplification of the GSIS function. MAF BZIP transcription factor B (*MAFB*) alternatively regulates *INS* gene expression in human β cells (Russell et al., 2020). Metabolic shift triggered by weaning enhances GSIS (Jacovetti et al., 2015; Stolovich-Rain et al., 2015). SIX2 is important for sustaining key β-cell lineage and metabolic genes (Arda et al., 2016; Velazco-Cruz et al., 2020a; Bevacqua et al., 2021). ATP and other nutritions, including glucose, have been reported to enhance the maturity of juvenile/neonate β cells in rodents (Rorsman et al., 1989; Hellerstrom and Swenne, 1991). We have previously demonstrated that estrogen-related receptor-γ (ERRγ) and related metabolic genes are upregulated during postnatal development in mouse islets (Yoshihara et al., 2016). ERRγ directly regulates gene clusters related to mitochondrial pyruvate metabolism, tricarboxylic acid (TCA) cycle, and oxidative phosphorylation (OxPhos), causing enhanced glucose-stimulated ATP production and insulin secretion (Yoshihara et al., 2016; Ahmadian et al., 2018; Fan et al., 2018). These metabolic reprogramming may be postnatally coordinated by the non-canonical WNT pathway (ncWNT4) (Bader et al., 2016; Yoshihara et al., 2020). Flattop (Fltp), a receptor of Wnt4, is upregulated postnatally in murine β cells, and Wnt4 enhances the maturation of β cells in mice and humans. We demonstrated that Wnt4 expression is significantly lower in murine neonatal islets or hPSC-derived β-like cells than that in murine adult β cells or primary adult human islets (Bader et al., 2016; Yoshihara et al., 2016; Yoshihara et al., 2020). Additionally, Wnt4 treatment enhances oxidative features and GSIS function in hPSC-derived 3D-structured human islet-like organoids (HILOs). These studies indicate that not only lineage commitment but also metabolic adaptation is important for enhancing the functional maturation of hPSC-derived β-like cells or islets. Other lineage-dependent transcriptional factors (LDTFs) and signal-dependent transcriptional factors (SDTFs), which collaboratively modulate functional maturation, have been discussed elsewhere (Wortham and Sander, 2021). The β-cell function is enhanced through aging in rodents, and p16 plays a key role in reducing cell proliferation and enhancing GSIS function. Circadian rhythms acquired during development also play an important role in β-cell maturation (Alvarez-Dominguez et al., 2020). Recent advances in single-cell technologies have enabled recapturing of the gene clusters involved in the differentiation and *in vivo* maturation of hPSC-derived β-like cells (Veres et al., 2019; Augsornworawat et al., 2021; Balboa et al., 2022). Several key β-cell genes in hPSC-derived β-like cells, including *INS*, *MAFA*, *G6PC2,* and *UCN3*, have been observed to be expressed in equivalent amounts only after *in vivo* maturation (Veres et al., 2019; Augsornworawat et al., 2021; Balboa et al., 2022). From these studies, the key changes during functional β-cell maturation seem to be advanced lineage commitment, metabolic reprograming to a more oxidative status with efficient glucose utilization, cell cycle reduction, and adaptation to environmental physiological changes *in vivo*.

### Differences Between Primary Human Islets and hPSC-Derived Islets

Although there has been a better understanding of the pancreatic differentiation, islets maturation, and the technologies mimicking human organogenesis, there are many differences between the primary human islets supplied by the human donors and the generated hPSC-derived islets. Generally, hPSC-derived islets exhibit lower insulin production and GSIS fold changes than adult human primary islets (Pagliuca et al., 2014; Rezania et al., 2014; Velazco-Cruz et al., 2020b; Yoshihara et al., 2020). These functional differences may be insufficiently represented since the primary adult islets functionally differ among individuals, and the donor islets were typically shipped at 4 C overnight or a few days, which makes the condition of primary human islets unideal. This suggests that the functional differences between hPSC-derived islets and primary adult islets maybe even bigger than the ones described or thought of previously. In 2014, Hrvatin et al. (2014) revealed that the hPSC-derived β-like cells resemble human fetal β cells more transcriptionally rather than adult β cells. Remarkable technological advances have been made to create more glucose-responsive hPSC-derived β-like cells, which are capable of secreting insulin in response to high glucose, to produce mono-hormonal insulin-producing cells rather than poly-hormonal insulin-producing cells, and for improved differentiation efficacy to create insulin-producing cells to about ∼70%. The insulin production levels are significantly higher than those in previous protocols (Pagliuca et al., 2014; Rezania et al., 2014; Russ et al., 2015). Although considerable progress has been made in creating functional islets from hPSCs, there are still significant differences between hPSC-derived β-like cells and human primary β cells. Ca^2+^ influx, which is triggered by the closure of the K^ATP^ channel in response to ATP amplification typically induced by glucose metabolism in the mitochondria of hPSC-derived β-like cells, is much slower and abnormal compared with that in the primary human islets ^23,24,4^. This is linked to the slower and fewer insulin secretion responses in hPSC-derived β-like cells (Pagliuca et al., 2014; Rezania et al., 2014; Nair et al., 2019). Patch-clamp recordings revealed heterogeneity and aberrant ion current such as Na+ (Balboa et al., 2022). Aberrant metabolic features in hPSC-derived β-like cells or islets, such as glycolysis, TCA cycle, and OxPhos, were observed and demonstrated as the target pathways to improve GSIS (Yoshihara et al., 2016; Nair et al., 2019; Davis et al., 2020; Balboa et al., 2022). It has been observed that longer culturing time of hPSC-derived islets at the maturation stage *in vitro* improves some maturation marker gene expression and GSIS; however, it has been identified that *in vivo* engraftment for 6 months is necessary to promote further maturation (Veres et al., 2019; Augsornworawat et al., 2020; Balboa et al., 2022). There remain several missing factors to understand how pancreatic islets adapt to the physiological environment during functional maturation. These factors should be considered to further improve the generation of functional human islets from hPSCs (Figure 1).

### Current Progress and Missing Factors for Generating Fully Functional Human Islets

#### Dynamic Metabolic Circulation

Blood glucose levels are elevated postnatally triggered by weaning owing to the alternation of nutritional sources from fat-rich breast milk to a carbohydrate-rich chow diet (Stolovich-Rain et al., 2015). Glucose has been identified as a maturation factor in rodent β cells (Hellerstrom and Swenne, 1991). Therefore, the current protocol for *in vitro* β-cell generation is intended to increase glucose concentration during differentiation from the PP stage to the β-cell maturation stage (Yoshihara et al., 2020). The bottleneck of glycolysis (Davis et al., 2020) or mitochondrial metabolism (Yoshihara et al., 2016) in hPSC-derived β-like cells may limit the glucose-induced maturation efficacy. Improving β-cell metabolism has been highlighted as the target for improving maturation. We have previously demonstrated that forced expression of estrogen-related receptor γ (ERRγ), a master regulator of mitochondrial gene expression, including pyruvate metabolism, the TCA cycle, and OxPhos improves GSIS in hPSC-derived β-like cells (Yoshihara et al., 2016). Reaggregation (Nair et al., 2019) or the ncWnt4/planar cell polarity (PCP) pathway (Bader et al., 2016; Yoshihara et al., 2020) enhances mitochondrial metabolism, which enhances GSIS function in hPSC-derived β-like cells. ncWnt/PCP effector Flattop (Fltp) expression increases during postnatal β cell maturation and is heterogeneously expressed in mature β cells. Fltp-positive (Fltp+) β cells are characterized by higher mitochondrial gene expression and activity (Bader et al., 2016). Wnt4, a component of ncWnt pathway increases during postnatal β cell maturation, and Wnt4 expression is lower in hPSC-derived β-like cells than that in the primary human β cells. Wnt4 stimulation enhances expression in mitochondrial gene expression and activity and GSIS function in hPSC-derived human islet-like organoids (HILOs). We have reported that ERRγ is a downstream factor of Wnt4 signaling to regulate mitochondrial gene expression and activity (Yoshihara et al., 2020). Declining mammalian target of rapamycin complex 1 (mTORC1) signaling from neonate to adult maturation has been observed (Ni et al., 2017; Jaafar et al., 2019; Helman et al., 2020). The constitutively active mTORC pathway in mouse β cells increases β-cell proliferation while reducing β cells’ maturation gene expressions such as *Ins1*, *Ucn3*, *Mafa,* and *G6pc2* and mitochondrial metabolic genes (Jaafar et al., 2019). mTORC1 enhances the responses of amino acid-stimulated insulin secretion, which increases insulin secretion at low glucose concentrations and reduces GSIS thresholds (Helman et al., 2020). In addition to mitochondria metabolism, glycolysis is impaired in hPSC-derived β-like cells, which limits the pyruvate and related fuel mitochondrial metabolite production. The enzymatic activity, but not the expression of glyceraldehyde 3-phosphate dehydrogenase (GAPDH) and phosphoglycerate kinase 1 (PGK1), which are important for the enzymatic conversion of glyceraldehyde 3-phosphate to 3-phosphoglycerate in glycolysis in hPSC-derived β-like cells, is defective (Davis et al., 2020). This bottleneck of enzymatic activity slows down the glycolysis activity, limiting the production of phosphoenolpyruvate to fuel ATP generation through the mitochondria. Cell-permeable intermediate metabolites such as methyl-succinate and methyl-pyruvates partially rescue the GSIS defect in hPSC-derived β-like cells. Since most of the hPSC-derived β-like cells generated *in vitro* present significantly lower maximum respiration of the mitochondria, efficient supply of the mitochondria and improvement of the mitochondria are some of the key features of immature hPSC-derived β-like cells.

Dynamic continuous nutritional exchange is the natural condition for endogenous functional β-like cells; however, it has not been replicated for *in vitro*-cultured hPSC-derived β-like cells. The current protocol relies on feeding nutrition-rich media during β-like cell differentiation every day or every other day. In this setting, glucose is utilized without refill for more than 24–48 h and waste such as lactate from the cells continues to accumulate in the culture condition. Lactate and pyruvate transporters, such as monocarboxylate transporter 1 (MCT1), are disallowance genes in functional β cells, and their expression has been observed in neonates or hPSC-derived β-like cells (Yoshihara et al., 2016). This notion raises the concern that accumulated lactate not only oxidizes the media but is also re-absorbed in the hPSC-derived β-like cells to interrupt the proper metabolic activity and differentiation in β cells. In addition, a recent study found that hPSC-derived islets are hyper-sensitive to pyruvate-induced insulin secretion, which typically is not observed in primary adult islets (Balboa et al., 2022). Aberrant pyruvate transporter expression may contribute to this abnormal phenotype of hPSC-derived islets. *In vivo*-mimicking continuous nutritional exchange by techniques such as microfluidic perfusion (Jun et al., 2019) may aid in improving the metabolic disorder in hPSC-derived β-like cells.

#### Oxygen Supply

In addition to nutrition, oxygen is a critical factor responsible for the functionality of β cells. Mature islets are surrounded by microvessels, which provide circulating oxygen tension (approximately 100 mmHg) (Jansson and Hellerstrom, 1983). It has been observed that *in vitro*-cultured human islet viability, especially hypoxia-induced necrosis in the center of islets, is improved by hyperoxia (approximately 350 mmHg) (Komatsu et al., 2016; Komatsu et al., 2017). Since there is no oxygen supply from microvessel *in vitro*-cultured hPSC-derived islets, current typical cell culture conditions, 5% CO_2_, 20% O_2_ (approximately 160 mmHg), may not be ideal for differentiation and maintenance. The inner cell mass of blastocyst is likely to maintain low O_2_ condition (approximately 5% O_2_) (Rodesch et al., 1992; Clark et al., 2006; Harvey, 2007; Lees et al., 2019). This is reasonable since hPSC metabolism is characterized by being highly dependent on glycolysis and up to 70% of glucose is converted to lactate (Lees et al., 2019). During differentiation and maturation, this metabolism is shifted to a more oxidative mitochondria-dependent process (Varum et al., 2011). High O_2_ levels (approximately 35% O_2_) promote the growth of *ex vivo*-cultured mouse pancreatic buds (Fraker et al., 2007). Similarly, rat pancreata cultured with high O_2_ (60–80% O_2_) promotes β-cell differentiation (Heinis et al., 2010). Mechanistically, hypoxia activates hypoxia-inducible factor 1α (HIF1α), which in turn activates the hairy and enhancer of split (HES1) to prevent β-cell differentiation (Heinis et al., 2010). High O_2_ (60% O_2_) exposure between the endoderm and EP stage facilitated differentiation of EPs by activating cWnt signaling and suppressing HIF1α and HES1 expression, which leads to the promotion of NGN3 expression in the insulin-producing cell differentiation model of mouse and human ES cells (Cechin et al., 2014; Hakim et al., 2014). In contrast, an excessive amount of O_2_ may increase the reactive oxygen species (ROS) and cause oxidative stress which triggers β-cell dysfunction (Robertson et al., 2004; Gerber and Rutter, 2017). Both hypoxia and oxidative stress/ROS induce thioredoxin-interacting protein (Txnip), a major cause of β-cell dysfunction in diabetes (Yoshihara et al., 2010a; Yoshihara et al., 2010b; Yoshihara et al., 2014; Yoshihara, 2020). Therefore, rather than a continuous supply of excessive O_2_, more dynamic regulation of O_2_ supply should be considered to improve the current protocol for the differentiation of the β cells. Monitoring the dynamic O_2_ supply will benefit the better differentiation and function of hPSC-derived β cells.

#### Fibroblasts, Pancreatic Exocrine, and Immune Cell Interactions

Islet microenvironments consist of pancreatic fibroblasts, acinar cells, vasculature cells, and immune cells such as macrophages. Increasing evidence suggests that exocrine acinar cells play a critical role in maintaining islet function and defects in exocrine link to the pathogenesis of some types of diabetes (Rickels et al., 2020; Chiou et al., 2021). The pancreatic organ-matched mesenchyme promotes self-renewal of the hPSC-derived PPs without further direct differentiation (Sneddon et al., 2012). These studies suggest that the islet microenvironment created by non-endocrine cells contributes to islet development and functional regulation. Further investigation of the spatiotemporal interaction between the endocrine cells and non-endocrine cells in islets is required to identify the key pathway that facilitates islet differentiation and physiological activity during fetal and postnatal development.

#### Peripheral Neuro-Vasculature System

It has been widely recognized that central and peripheral neurons play an important role in regulating the exocrine and endocrine pancreas (Buijs et al., 2001; Chandra and Liddle, 2013). Neural projections from the dorsal motor neuron (dMN) in the brain stem innervate the pancreas and regulate enzymatic and hormonal secretions in the exocrine and endocrine systems, respectively. Thyrosine hydroxylase (TH)-positive sympathetic nerves have been detected in the fetal pancreas starting from the early stages, such as 8 weeks post-conception (w.p.c) (Krivova et al., 2021). The sympathetic nerve innervation of mouse islets is reported to be a critical factor for proper islet architecture and function in a β-adrenergic dependent manner (Borden et al., 2013). Although how the innervation of peripheral nerves in human islets is important for organizing islet architecture, development, and function is still controversial or remains unknown, many receptors for neurotransmitters are expressed in human islets and regulate hormone secretion (Ahren, 2000; Burns et al., 2015; Rosario et al., 2016; Lin et al., 2021).

#### Paracrine Regulation

Islets are multicellular mini-organs consisting of differential hormone-expressing cells with paracrine hormone regulation (Huising, 2020). Α cells secrete glucagon and glucagon-like peptide-1 (GLP-1), which enhance insulin secretion in β cells. δ cells secrete somatostatin, which suppresses insulin secretion in β cells and glucagon secretion from α cells (Strowski et al., 2000). Insulin inhibits glucagon secretion by stimulating somatostatin secretion by sodium-glucose co-transporter-2 (SGLT2) (Vergari et al., 2019). *UCN3*, co-secreted with insulin, also stimulates somatostatin secretion from δ cells to enhance paracrine feedback regulation of β cells (van der Meulen et al., 2015). Although this paracrine hormone regulation plays an important role in proper insulin secretion in islets, the effect of paracrine signaling on islet differentiation and maturation has not yet been elucidated.

#### Organ–Organ Interaction

Islets are embedded in the exocrine pancreas while through the nervous innervation and vasculature network receiving the signals from outside the pancreas. The intestine plays a central role in regulating β-cell function through the enteroendocrine hormone secretion directly or via the vagus nerve–brain–pancreas axis. Adipokines and hepatokines also play an important role in β-cell function (El Ouaamari et al., 2013; Prentice et al., 2021). These signals from outside the pancreas influence the differentiation and maturation of islets remain poorly understood. Since the organs are simultaneously differentiated and maturate during fetal and postnatal development, it is possible that organ–organ interactions also play an important role in islet differentiation and maturation.

### Adapting Physiology

Adapting to physiological environmental stimuli is a hallmark of mature adult organs, which are broadly regulated by nuclear hormone receptor (NR) signaling (Evans and Mangelsdorf, 2014). NR, a superfamily of 48 transcriptional factors in humans, regulates gene expression in response to various hormones, lipids, and metabolites and plays a master role in development and physiology (Evans and Mangelsdorf, 2014). Many NRs are expressed in rodent and human islets and are related to the β-cell physiology in response to lipid metabolism (Chuang et al., 2008) (Table 1). Free fatty acids, such as palmitate, stimulate insulin secretion for the short term, whereas chronic exposure to high concentrations of free fatty acids induces compensatory β-cell expansion and eventually leads to β-cell growth arrest and apoptosis. Nuclear receptor subfamily 4 group A member 1 (NR4A1) is translocated to the nucleus in response to free fatty acids and plays a key role in lipotoxicity-induced β-cell apoptosis (Susini et al., 1998; Roche et al., 1999; Briand et al., 2012; Yu et al., 2015; Close et al., 2019). Mechanistically, NR4A1 binds to Foxo1 at the *MafA* promoter region and suppresses its expression to reduce *insulin* transcription (Briand et al., 2012). NR4A1 is required for Nkx6-1-mediated β-cell mass expansion (Tessem et al., 2014). Cholesterol sensor Liver X receptor (LXR; LXRα and LXRβ) stimulates GSIS and insulin biosynthesis (Efanov et al., 2004; Zitzer et al., 2006). LXRβKO mice exhibit a glucose-intolerant phenotype secondary to blunted GSIS *in vivo* (Gerin et al., 2005; Zitzer et al., 2006). Peroxisome proliferator-activated receptors (PPARs; PPARγ, PPARα, and PPARδ) broadly regulate fatty acid oxidation, thereby enhancing GSIS and reducing lipotoxic effects in β cells. Estrogen receptors (ERs; ERα and ERβ) also protect against lipotoxicity, especially in females. Testosterone-mediated andorogen receptor (AR) signaling enhances GSIS and glucagon-like peptide-1 (GLP-1)-mediated insulin secretion in males (Navarro et al., 2016). Vitamin D receptor (VDR) regulates SWI/SNF chromatin remodeling together with bromodomain-containing protein 7 (BRD7) and BRD9, and protection from cytokines induces dedifferentiation of β cells in rodents and hPSC-derived β-like cells (Wei et al., 2018). Estrogen-related receptor γ (ERRγ) regulates mitochondrial gene expression related to the TCA cycle and OxPhos (Yoshihara et al., 2016). Farnesoid X receptors (FXRs; FXRα and FXRβ) regulate *Ins* (Renga et al., 2010), adenylyl cyclase 8 (*Adcy8*) (Kong et al., 2019a), transient receptor potential ankyrin 1 (*Trpa1*) (Kong et al., 2019b), and GLP-1 receptor (*Glp1r*) (Kong et al., 2021) gene expression and protect from lipotoxicity (Popescu et al., 2010) in the rodents. Glucocorticoids and steroids are known to induce hyperglycemia, which may cause diabetes. Glucocorticoid receptor (GR) signaling through dexamethasone was initially believed to have a negative impact on islet physiology by reducing insulin synthesis and insulin secretion and inducing apoptosis in murine β cells (Philippe and Missotten, 1990; Goodman et al., 1996; Ranta et al., 2006; Reich et al., 2012; Aylward et al., 2021). In contrast, GR plays an essential role in human fetal and adult islet development by controlling β-cell transcriptomes, such as *PDX-1*, *GCK*, *GLUT2*, *SIX2,* and *SIX3*(*Gesina et al., 2006*; *Matthews and Hanley, 2011*; *Ghazalli et al., 2018*)*.* Thyroid hormone receptors (TRs; TRα and TRβ) regulate *MafA* expression during postnatal rat β-cell maturation (Aguayo-Mazzucato et al., 2013). Hypothyroidism impairs hPSC-PP maturation in mice suggesting the importance of TRs signaling *in vivo* functional β-cell maturation (Bruin et al., 2016). Circadian rhythms regulate β-cell maturation and physiology (Marcheva et al., 2010; Perelis et al., 2015; Alvarez-Dominguez et al., 2020). The clock protein REV-ERVα regulates the circadian rhythms in β cells, and downregulation of REV-ERVα impairs time-dependent GSIS (Vieira et al., 2012). REV-ERVα may be an important physiological regulator of β cells in time-restricted feeding or preventing pathogenesis of T2D by modulating β-cell rhythmic transcriptional regulation by adapting to environmental changes over time (Petrenko et al., 2020; Brown et al., 2021). Hepatic nuclear receptor 4-α (HNF4α) is responsible for maturity-onset diabetes of the young 1 (MODY1) (Yamagata et al., 1996). Although NRs integrate gene regulation and cell function to adapt to environmental physiological stimulation, the contribution of NRs to functional maturation to adapt to environmental stimulation in islets is largely unknown. Understanding how these factors cooperatively regulate β-cell differentiation and physiological adaptation may improve generation of a physiological level of functional β cells.

**TABLE 1:** Physiological role of nuclear receptors in functional β cells.

Common name	Symbol	Abbreviation	Ligands	Function	Human islets (FPKM)	Mouse islets (FPKM)	References
Thyroid hormone receptor-α	NR1A1	TRα	Thyroid hormone	Upregulation MAFA, GCK gene expression. Enhances GSIS, insulin production	>10	>20	Aguayo-Mazzucato et al. (2013)
Thyroid hormone receptor-β	NR1A1	TRβ		Upregulation MAFA, GCK gene expression. Enhances GSIS, insulin production	>1	>2	Aguayo-Mazzucato et al. (2013)
Retinoic acid receptor-α	NR1B1	RARα	Vitamin A, retinoic acids	Endocrine differentiation. Regulation of β-cell mass	>5	>5	Brun et al. (2015), Matthews et al. (2004), Stafford and Prince, (2002)
Retinoic acid receptor-β	NR1B2	RARβ		Endocrine differentiation	>0.5	>0.4	Perez et al. (2013), Fedullo et al. (2021), Stafford and Prince, (2002)
Retinoic acid receptor-γ	NR1B3	RARγ		Endocrine differentiation	>1	>10	Fedullo et al. (2021), Stafford and Prince, (2002)
Peroxisome proliferator-activated receptor-α	NR1C1	PPARα	Fatty acids, prostaglandins	Enhances fatty acid oxidation and insulin secretion	>3	>0.3	Lalloyer et al. (2006), Zhou et al. (1998)
Peroxisome proliferator-activated receptor- δ	NR1C2	PPARδ		Enhances fatty acid oxidation and insulin secretion	>6	>12	Tang et al. (2013)
Peroxisome proliferator-activated receptor-γ	NR1C3	PPARγ		Enhances fatty acid oxidation and insulin secretion	>0.3	>5	Dubois et al. (2000), Rosen et al. (2003)
Rev-ErbAα	NR1D1	Rev-ErbAα	Heme	Circadian oscillation	>14	>30	Saini et al. (2016), Pulimeno et al. (2013)
Rev-ErbAα	NR1D2	Rev-ErbAβ		Circadian oscillation	>20	>30	Saini et al. (2016), Pulimeno et al. (2013)
RAR-related orphan receptor-α	NR1F1	RORα	Cholesterol, retinoic acids	Suppresses GSIS. Circadian oscillation	>3	>1	Muhlbauer et al. (2013)
RAR-related orphan receptor-β	NR1F2	RORβ		Suppresses GSIS. Circadian oscillation	>1	>0.01	Taneera et al. (2019), Muhlbauer et al. (2013)
RAR-related orphan receptor-γ	NR1F3	RORγ		Suppresses GSIS. Circadian oscillation	>7	>10	Taneera et al. (2019), Muhlbauer et al. (2013)
Liver X receptor-β	NR1H2	LXRβ	Cholesterol	Enhances glycerol/fatty acids cycling	>13	>30	Efanov et al. (2004), Zitzer et al. (2006), Gerin et al. (2005)
Liver X receptor-α	NR1H3	LXRα		Enhances glycerol/fatty acids cycling	>2	>5	Efanov et al. (2004)
Farnesoid X receptor-α	NR1H4	FXRα	Bile acids	Upregulation of INS, GLP1R gene expression	>1	>9	Renga et al. (2010), Kong et al. (2019a), Kong et al. (2019b), Dufer et al. (2012), Kong et al. (2021)
Farnesoid X receptor-β	NR1H5	FXRβ		-	-	>0.01	-
Vitamin D receptor	NR1I1	VDR	Vitamin D	Suppresses inflammation and prevents cytokine induced β-cell dedifferentiation. Modulation of BAF complex	>3	>40	Wei et al. (2018)
Pregnane X receptor	NR1I2	PXR	Xenobiotics	Unclear	>0.01	>0.05	-
Constitutive androstane receptor	NR1I3	CAR	Androstane	Unclear	>0.1	>0.03	-
Hepatocyte nuclear factor-4-α	NR2A1	HNF4α	Fatty acids	Responsible gene for MODY1	>3	>10	Yamagata et al. (1996)
Hepatocyte nuclear factor-4-γ	NR2A2	HNF4γ		Pancreatic differentiation	>0.2	>2	Boj et al. (2001)
Retinoid X receptor-α	NR2B1	RXRα	Retinoic acids	Pancreatic differentiation	>10	>12	Kane et al. (2010), Kadison et al. (2001)
				Attenuates GSIS			
Retinoid X receptor-β	NR2B2	RXRβ		Pancreatic differentiation	>18	>18	Kane et al. (2010), Kadison et al. (2001)
				Attenuates GSIS			
Retinoid X receptor-γ	NR2B3	RXRγ		Pancreatic differentiation	>5	>0.1	Kane et al. (2010), Kadison et al. (2001)
				Attenuates GSIS			
Testicular receptor 2	NR2C1	TR2	-	Unclear	>3	>3	-
Testicular receptor 4	NR2C2	TR4		Unclear	>6	>6	-
Homolog of the Drosophila tailless gene	NR2E1	TLX	-	Enhances β-cell proliferation	>0.01	-	Shi et al. (2019), Shi et al. (2015)
Photoreceptor cell-specific nuclear receptor	NR2E3	PNR	-	Unclear	>0.01	>0.01	-
Chicken ovalbumin upstream promoter-α	NR2F1	COUP-TFα	-	Negatively regulates the mouse INS2 gene	>0.5	>0.3	Zhang et al. (2017), Boutant et al. (2012), Qin et al. (2010), Perilhou et al. (2008), Bardoux et al. (2005)
Chicken ovalbumin upstream promoter-β	NR2F2	COUP-TFβ		Positively regulates β-cell proliferation. Islet tumorigenesis	>6	>1	Qin et al. (2010), Boutant et al. (2012)
Chicken ovalbumin upstream promoter-γ	NR2F6	COUP-TFγ		Unclear	>25	>23	-
Estrogen receptor-α	NR3A1	ERα	Estrogens	Regulates insulin synthesis. Suppresses lipid synthesis	>1	>0.03	Alonso-Magdalena et al. (2008), Wong et al. (2010), Tiano et al. (2011)
Estrogen receptor-β	NR3A2	ERβ		Regulates insulin synthesis. Suppresses lipid synthesis	>0.01	>0.02	Tiano et al. (2011), Wong et al. (2010), Alonso-Magdalena et al. (2008)
Estrogen-related receptor-α	NR3B1	ERRα		Unclear/possibly upregulates mitochondrial gene expression and enhances oxidative metabolism	>23	>10	-
Estrogen-related receptor-β	NR3B2	ERRβ		Unclear	>0.1	>0.1	-
Estrogen-related receptor-γ	NR3B3	ERRγ		Upregulates mitochondrial gene expression and enhances oxidative metabolism and postnatal maturation	>2	>2	Yoshihara et al. (2016), Yoshihara et al. (2020)
Glucocorticoid receptor	NR3C1	GR	Cortisol	Promotes pancreatic differentiation. Induces apoptosis	>14	>17	Aylward et al. (2021), Ghazalli et al. (2018), Reich et al. (2012), Matthews and Hanley, (2011), Ranta et al. (2006), Gesina et al. (2006), Shapiro et al. (2000)
Mineralocorticoid receptor	NR3C2	MR	Aldosterone	Enhances insulin secretion through α-cell GLP1 secretion	>5	>2.5	Goto et al. (2019)
Progesterone receptor	NR3C3	PR	Progesterone	Negatively regulates β-cell proliferation	>2	>0.05	Picard et al. (2002)
Androgen receptor	NR3C4	AR	Testosterone	Enhances GSIS	>0.2	>0.1	Navarro et al. (2016)
Nerve growth factor IB	NR4A1	NGFIB	-	Positively and negatively regulates β-cell proliferation	>2	>15	Yu et al. (2015), Tessem et al. (2014), Briand et al. (2012)
Nuclear receptor related 1	NR4A2	NURR1	-	Positively regulates β-cell proliferation	>1	>1	Close et al. (2019)
Neuron-derived orphan receptor 1	NR4A3	NOR1	-	Positively and negatively regulates β-cell proliferation	>0.5	>0.05	Tessem et al. (2014), Briand et al. (2012), Close et al. (2019)
Steroidogenic factor 1	NR5A1	SF-1	Phosphatidylinositols	Unclear	-	-	-
Liver receptor homolog-1	NR5A2	LRH-1		Pancreas organogenesis. Protects from stress-induced β-cell apoptosis	>2	>0.4	Hale et al. (2014), Baquie et al. (2018), Cobo-Vuilleumier et al. (2018)
Germ cell nuclear factor	NR6A1	GCNF	-	Unclear	>0.2	>2.5	-
Dosage-sensitive sex reversal, adrenal hypoplasia critical region, on chromosome X, gene 1	NR0B1	DAX1	-	Unclear	>17	-	-
Small heterodimer partner	NR0B2	SHP	-	Negatively regulates β-cell survival	>7	>0.01	Noh et al. (2013), Seo et al. (2008), Park et al. (2007), Suh et al. (2004)

## Conclusion

I discussed the existing limitations of the technology to generate human islets from pluripotent stem cells. The current protocol is generated on the basis of lineage specification; however, sufficient number of integrated functional maturation pathways are not there. The challenge remains to create cells with protocols within a few weeks of additional maturation, which exhibit equal functionality with primary human islets in terms of 1) amount of insulin secretion, 2) speed and accuracy of response to glucose, 3) transcriptome, and 4) controlled differentiation to individual endocrine cell types by integrating the new pathways adapting to environmental physiological stimulation. The key challenge in the field is that maturation may not only be coordinated by single signaling but corporately regulates by multiple signaling as the environmental senor switch (Figure 1). Additionally, the required signaling may not be constitutively activated or suppressed; however, it is dynamically regulated by adapting to environmental physiological situations to work flexibly and properly in the spatiotemporal physiological situation. The future challenge of developing new technology should focus on dissecting many differential signaling and transcriptional networks that are involved in postnatal functional maturation and integrating how those signaling pathways synergistically regulate islet maturation. The robustness of scalability, consistency of high quality, and less batch-to-batch generation of different human islets *in vitro* should be continuously improved. One important notion for adapting to environmental signals is the duration and timing of the stimulation of those signals. The current protocol generates glucose-responsive hPSC-derived β-like cells or islets within 4–10 weeks of culturing *in vitro*. However, it takes approximately 40 weeks of differentiation and a few years of postnatal functional islet maturation in human body. This significant gap in the developmental time may need to be considered adapting to environmental cues in functional human islet organogenesis. A deeper understanding of islet differentiation and maturation is critical for generating fully functional human islets *in vitro*. Integrating the microenvironmental cues that mimic *in vivo* situations may help in further improving the protocol.

## Data Availability

The original contributions presented in the study are included in the article/Supplementary Material, further inquiries can be directed to the corresponding author.

## References

[B1] Aguayo-Mazzucato C. Koh A. El Khattabi I. Li W.-C. Toschi E. Jermendy A. (2011). Mafa Expression Enhances Glucose-Responsive Insulin Secretion in Neonatal Rat Beta Cells. Diabetologia 54, 583–593. 10.1007/s00125-010-2026-z 21190012 PMC3047400

[B2] Aguayo-Mazzucato C. Zavacki A. M. Marinelarena A. Hollister-Lock J. El Khattabi I. Marsili A. (2013). Thyroid Hormone Promotes Postnatal Rat Pancreatic β-Cell Development and Glucose-Responsive Insulin Secretion through MAFA. Diabetes 62, 1569–1580. 10.2337/db12-0849 23305647 PMC3636623

[B3] Ahmadian M. Liu S. Reilly S. M. Hah N. Fan W. Yoshihara E. (2018). ERRγ Preserves Brown Fat Innate Thermogenic Activity. Cel Rep. 22, 2849–2859. 10.1016/j.celrep.2018.02.061 PMC5884669 29539415

[B4] Ahrén B. (2000). Autonomic Regulation of Islet Hormone Secretion - Implications for Health and Disease. Diabetologia 43, 393–410. 10.1007/s001250051322 10819232

[B5] Alonso-Magdalena P. Ropero A. B. Carrera M. P. Cederroth C. R. Baquié M. Gauthier B. R. (2008). Pancreatic Insulin Content Regulation by the Estrogen Receptor ERα. PLoS One 3, e2069. 10.1371/journal.pone.0002069 18446233 PMC2323613

[B6] Alvarez-Dominguez J. R. Donaghey J. Rasouli N. Kenty J. H. R. Helman A. Charlton J. (2020). Circadian Entrainment Triggers Maturation of Human *In Vitro* Islets. Cell Stem Cell 26, 108–122. e10. 10.1016/j.stem.2019.11.011 31839570

[B7] Alvarez-Dominguez J. R. Melton D. A. (2021). Cell Maturation: Hallmarks, Triggers, and Manipulation. Cell 185, 235. 10.1016/j.cell.2021.12.012 PMC8792364 34995481

[B8] Arda H. E. Li L. Tsai J. Torre E. A. Rosli Y. Peiris H. (2016). Age-Dependent Pancreatic Gene Regulation Reveals Mechanisms Governing Human β Cell Function. Cel Metab. 23, 909–920. 10.1016/j.cmet.2016.04.002 PMC4864151 27133132

[B9] Artner I. Hang Y. Mazur M. Yamamoto T. Guo M. Lindner J. (2010). MafA and MafB Regulate Genes Critical to β-Cells in a Unique Temporal Manner. Diabetes 59, 2530–2539. 10.2337/db10-0190 20627934 PMC3279542

[B169] Assady S. Maor G. Amit M. Itskovitz-Eldor J. Skorecki K. L. Tzukerman M. (2001). Insulin Production by Human Embyonic Stem Cells. Diabetes 50 (8), 1691–7. 10.2337/diabetes.50.8.1691 11473026

[B10] Augsornworawat P. Maxwell K. G. Velazco-Cruz L. Millman J. R. (2020). Single-Cell Transcriptome Profiling Reveals β Cell Maturation in Stem Cell-Derived Islets after Transplantation. Cel Rep. 32, 108067. 10.1016/j.celrep.2020.108067 PMC7491368 32846125

[B11] Augsornworawat P. Maxwell K. G. Velazco-Cruz L. Millman J. R. (2021). Single-cell Transcriptome Profiling Reveals β Cell Maturation in Stem Cell-Derived Islets after Transplantation. Cel Rep. 34, 108850. 10.1016/j.celrep.2021.108850 PMC8020860 33691098

[B12] Aylward A. Okino M.-L. Benaglio P. Chiou J. Beebe E. Padilla J. A. (2021). Glucocorticoid Signaling in Pancreatic Islets Modulates Gene Regulatory Programs and Genetic Risk of Type 2 Diabetes. Plos Genet. 17, e1009531. 10.1371/journal.pgen.1009531 33983929 PMC8183998

[B13] Bader E. Migliorini A. Gegg M. Moruzzi N. Gerdes J. Roscioni S. S. (2016). Identification of Proliferative and Mature β-cells in the Islets of Langerhans. Nature 535, 430–434. 10.1038/nature18624 27398620

[B14] Balboa D. Barsby T. Lithovius V. Saarimaki-Vire J. Omar-Hmeadi M. Dyachok O. (2022). Functional, Metabolic and Transcriptional Maturation of Human Pancreatic Islets Derived from Stem Cells. Nat. Biotechnol. 10.1038/s41587-022-01219-z PMC9287162 35241836

[B15] Baquié M. St-Onge L. Kerr-Conte J. Cobo-Vuilleumier N. Lorenzo P. I. Jimenez Moreno C. M. (2018). The Liver Receptor Homolog-1 (LRH-1) Is Expressed in Human Islets and Protects β-cells against Stress-Induced Apoptosis. Hum. Mol. Genet. 27, 406. 10.1093/hmg/ddx402 29186426

[B16] Bardoux P. Zhang P. Flamez D. Perilhou A. Lavin T. A. Tanti J.-F. (2005). Essential Role of Chicken Ovalbumin Upstream Promoter-Transcription Factor II in Insulin Secretion and Insulin Sensitivity Revealed by Conditional Gene Knockout. Diabetes 54, 1357–1363. 10.2337/diabetes.54.5.1357 15855320

[B17] Beckman J. A. Paneni F. Cosentino F. Creager M. A. (2013). Diabetes and Vascular Disease: Pathophysiology, Clinical Consequences, and Medical Therapy: Part II. Eur. Heart J. 34, 2444–2452. 10.1093/eurheartj/eht142 23625211

[B18] Bevacqua R. J. Lam J. Y. Peiris H. Whitener R. L. Kim S. Gu X. (2021). SIX2 and SIX3 Coordinately Regulate Functional Maturity and Fate of Human Pancreatic β Cells. Genes Dev. 35, 234–249. 10.1101/gad.342378.120 33446570 PMC7849364

[B19] Boj S. F. Párrizas M. Maestro M. A. Ferrer J. (2001). A Transcription Factor Regulatory Circuit in Differentiated Pancreatic Cells. Proc. Natl. Acad. Sci. U.S.A. 98, 14481–14486. 10.1073/pnas.241349398 11717395 PMC64707

[B20] Borden P. Houtz J. Leach S. D. Kuruvilla R. (2013). Sympathetic Innervation during Development Is Necessary for Pancreatic Islet Architecture and Functional Maturation. Cel Rep. 4, 287–301. 10.1016/j.celrep.2013.06.019 PMC3740126 23850289

[B21] Boutant M. Ramos O. H. P. Tourrel-Cuzin C. Movassat J. Ilias A. Vallois D. (2012). COUP-TFII Controls Mouse Pancreatic β-Cell Mass through GLP-1-β-Catenin Signaling Pathways. PLoS One 7, e30847. 10.1371/journal.pone.0030847 22292058 PMC3265526

[B22] Briand O. Helleboid-Chapman A. Ploton M. Hennuyer N. Carpentier R. Pattou F. (2012). The Nuclear Orphan Receptor Nur77 Is a Lipotoxicity Sensor Regulating Glucose-Induced Insulin Secretion in Pancreatic β-Cells. Mol. Endocrinol. 26, 399–413. 10.1210/me.2011-1317 22301783 PMC5417130

[B23] Brown M. R. Sen S. K. Mazzone A. Her T. K. Xiong Y. Lee J. H. (2021). Time-restricted Feeding Prevents Deleterious Metabolic Effects of Circadian Disruption through Epigenetic Control of β Cell Function. Sci. Adv. 7, eabg6856. 10.1126/sciadv.abg6856 34910509 PMC8673777

[B24] Bruin J. E. Saber N. O’Dwyer S. Fox J. K. Mojibian M. Arora P. (2016). Hypothyroidism Impairs Human Stem Cell-Derived Pancreatic Progenitor Cell Maturation in Mice. Diabetes 65, 1297–1309. 10.2337/db15-1439 26740603

[B25] Brun P. J. Grijalva A. Rausch R. Watson E. Yuen J. J. Das B. C. (2015). Retinoic Acid Receptor Signaling Is Required to Maintain Glucose‐stimulated Insulin Secretion and β‐cell Mass. FASEB j. 29, 671–683. 10.1096/fj.14-256743 25389133 PMC4314234

[B26] Buijs R. M. Chun S. J. Niijima A. Romijn H. J. Nagai K. (2001). Parasympathetic and Sympathetic Control of the Pancreas: a Role for the Suprachiasmatic Nucleus and Other Hypothalamic Centers that Are Involved in the Regulation of Food Intake. J. Comp. Neurol. 431, 405–423. 10.1002/1096-9861(20010319)431:4<405::aid-cne1079>3.0.co;2-d 11223811

[B27] Burns S. M. Vetere A. Walpita D. Dančík V. Khodier C. Perez J. (2015). High-throughput Luminescent Reporter of Insulin Secretion for Discovering Regulators of Pancreatic Beta-Cell Function. Cel Metab. 21, 126–137. 10.1016/j.cmet.2014.12.010 25565210

[B28] Cayabyab F. Nih L. R. Yoshihara E. (2021). Advances in Pancreatic Islet Transplantation Sites for the Treatment of Diabetes. Front. Endocrinol. 12, 732431. 10.3389/fendo.2021.732431 PMC8473744 34589059

[B29] Cechin S. Álvarez-Cubela S. Giraldo J. A. Molano R. D. Villate S. Ricordi C. (2014). Influence of *In Vitro* and *In Vivo* Oxygen Modulation on β Cell Differentiation from Human Embryonic Stem Cells. Stem Cell Transl Med 3, 277–289. 10.5966/sctm.2013-0160 PMC3952934 24375542

[B30] Chandra R. Liddle R. A. (2013). Modulation of Pancreatic Exocrine and Endocrine Secretion. Curr. Opin. Gastroenterol. 29, 517–522. 10.1097/mog.0b013e3283639326 23817137 PMC4114933

[B31] Chiou J. Geusz R. J. Okino M.-L. Han J. Y. Miller M. Melton R. (2021). Interpreting Type 1 Diabetes Risk with Genetics and Single-Cell Epigenomics. Nature 594, 398–402. 10.1038/s41586-021-03552-w 34012112 PMC10560508

[B32] Cho N. H. Shaw J. E. Karuranga S. Huang Y. Da Rocha Fernandes J. D. Ohlrogge A. W. (2018). IDF Diabetes Atlas: Global Estimates of Diabetes Prevalence for 2017 and Projections for 2045. Diabetes Res. Clin. Pract. 138, 271–281. 10.1016/j.diabres.2018.02.023 29496507

[B33] Chuang J.-C. Cha J.-Y. Garmey J. C. Mirmira R. G. Joyce J. J. J. (2008). Research Resource: Nuclear Hormone Receptor Expression in the Endocrine Pancreas. Mol. Endocrinol. 22, 2353–2363. 10.1210/me.2007-0568 18669644 PMC2582538

[B34] Clark A. R. Stokes Y. M. Lane M. Thompson J. G. (2006). Mathematical Modelling of Oxygen Concentration in Bovine and Murine Cumulus-Oocyte Complexes. Reproduction 131, 999–1006. 10.1530/rep.1.00974 16735539

[B35] Close A.-F. Dadheech N. Villela B. S. Rouillard C. Buteau J. (2019). The Orphan Nuclear Receptor Nor1/Nr4a3 Is a Negative Regulator of β-cell Mass. J. Biol. Chem. 294, 4889–4897. 10.1074/jbc.ra118.005135 30696767 PMC6442030

[B36] Cobo-Vuilleumier N. Lorenzo P. I. Rodríguez N. G. Herrera Gómez I. d. G. Fuente-Martin E. López-Noriega L. (2018). LRH-1 Agonism Favours an Immune-Islet Dialogue Which Protects against Diabetes Mellitus. Nat. Commun. 9, 1488. 10.1038/s41467-018-03943-0 29662071 PMC5902555

[B37] Creager M. A. Lüscher T. F. Cosentino F. Beckman J. A. (2003). Diabetes and Vascular Disease. Circulation 108, 1527–1532. 10.1161/01.cir.0000091257.27563.32 14504252

[B38] D'Amour K. A. Agulnick A. D. Eliazer S. Kelly O. G. Kroon E. Baetge E. E. (2005). Efficient Differentiation of Human Embryonic Stem Cells to Definitive Endoderm. Nat. Biotechnol. 23, 1534–1541. 10.1038/nbt1163 16258519

[B39] D'Amour K. A. Bang A. G. Eliazer S. Kelly O. G. Agulnick A. D. Smart N. G. (2006). Production of Pancreatic Hormone-Expressing Endocrine Cells from Human Embryonic Stem Cells. Nat. Biotechnol. 24, 1392–1401. 10.1038/nbt1259 17053790

[B40] Davis J. C. Alves T. C. Helman A. Chen J. C. Kenty J. H. Cardone R. L. (2020). Glucose Response by Stem Cell-Derived β Cells *In Vitro* Is Inhibited by a Bottleneck in Glycolysis. Cel Rep. 31, 107623. 10.1016/j.celrep.2020.107623 PMC7433758 32402282

[B41] Dhawan S. Tschen S.-I. Zeng C. Guo T. Hebrok M. Matveyenko A. (2015). DNA Methylation Directs Functional Maturation of Pancreatic β Cells. J. Clin. Invest. 125, 2851–2860. 10.1172/jci79956 26098213 PMC4563682

[B42] Dubois M. Pattou F. Kerr-Conte J. Gmyr V. Vandewalle B. Desreumaux P. (2000). Expression of Peroxisome Proliferator-Activated Receptor γ (PPARγ) in normal Human Pancreatic Islet Cells. Diabetologia 43, 1165–1169. 10.1007/s001250051508 11043863

[B43] Düfer M. Hörth K. Wagner R. Schittenhelm B. Prowald S. Wagner T. F. J. (2012). Bile Acids Acutely Stimulate Insulin Secretion of Mouse β-Cells via Farnesoid X Receptor Activation and KATP Channel Inhibition. Diabetes 61, 1479–1489. 10.2337/db11-0815 22492528 PMC3357280

[B44] Efanov A. M. Sewing S. Bokvist K. Gromada J. (2004). Liver X Receptor Activation Stimulates Insulin Secretion via Modulation of Glucose and Lipid Metabolism in Pancreatic Beta-Cells. Diabetes 53 (Suppl. 3), S75–S78. 10.2337/diabetes.53.suppl_3.s75 15561926

[B45] El Ouaamari A. Kawamori D. Dirice E. Liew C. W. Shadrach J. L. Hu J. (2013). Liver-derived Systemic Factors Drive β Cell Hyperplasia in Insulin-Resistant States. Cell Rep 3, 401–410. 10.1016/j.celrep.2013.01.007 23375376 PMC3655439

[B46] Evans R. M. Mangelsdorf D. J. (2014). Nuclear Receptors, RXR, and the Big Bang. Cell 157, 255–266. 10.1016/j.cell.2014.03.012 24679540 PMC4029515

[B47] Fan W. He N. Lin C. S. Wei Z. Hah N. Waizenegger W. (2018). ERRγ Promotes Angiogenesis, Mitochondrial Biogenesis, and Oxidative Remodeling in PGC1α/β-Deficient Muscle. Cel Rep. 22, 2521–2529. 10.1016/j.celrep.2018.02.047 PMC5860878 29514081

[B48] Fedullo A. L. Schiattarella A. Morlando M. Raguzzini A. Toti E. De Franciscis P. (2021). Mediterranean Diet for the Prevention of Gestational Diabetes in the Covid-19 Era: Implications of Il-6 in Diabesity. Int. J. Mol. Sci. 22. 10.3390/ijms22031213 PMC7866163 33530554

[B49] Fraker C. A. Álvarez S. Papadopoulos P. Giraldo J. Gu W. Ricordi C. (2007). Enhanced Oxygenation Promotes β-Cell Differentiation *In Vitro* . Stem Cells 25, 3155–3164. 10.1634/stemcells.2007-0445 17761759

[B50] Gerber P. A. Rutter G. A. (2017). The Role of Oxidative Stress and Hypoxia in Pancreatic Beta-Cell Dysfunction in Diabetes Mellitus. Antioxid. Redox Signaling 26, 501–518. 10.1089/ars.2016.6755 PMC5372767 27225690

[B51] Gerin I. Dolinsky V. W. Shackman J. G. Kennedy R. T. Chiang S.-H. Burant C. F. (2005). LXRβ Is Required for Adipocyte Growth, Glucose Homeostasis, and β Cell Function. J. Biol. Chem. 280, 23024–23031. 10.1074/jbc.m412564200 15831500

[B52] Gesina E. Blondeau B. Milet A. Le Nin I. Duchene B. Czernichow P. (2006). Glucocorticoid Signalling Affects Pancreatic Development through Both Direct and Indirect Effects. Diabetologia 49, 2939–2947. 10.1007/s00125-006-0449-3 17001468 PMC1885455

[B53] Ghazalli N. Wu X. Walker S. Trieu N. Hsin L.-Y. Choe J. (2018). Glucocorticoid Signaling Enhances Expression of Glucose-Sensing Molecules in Immature Pancreatic Beta-like Cells Derived from Murine Embryonic Stem Cells *In Vitro* . Stem Cell Develop. 27, 898–909. 10.1089/scd.2017.0160 PMC6029647 29717618

[B54] Goodman P. A. Medina-Martinez O. Fernandez-Mejia C. (1996). Identification of the Human Insulin Negative Regulatory Element as a Negative Glucocorticoid Response Element. Mol. Cell Endocrinol. 120, 139–146. 10.1016/0303-7207(96)03830-0 8832573

[B55] Goto R. Kondo T. Ono K. Kitano S. Miyakawa N. Watanabe T. (2019). Mineralocorticoid Receptor May Regulate Glucose Homeostasis through the Induction of Interleukin-6 and Glucagon-like Peptide-1 in Pancreatic Islets. J. Clin. Med. 8. 10.3390/jcm8050674 PMC6571682 31091693

[B56] Hakim F. Kaitsuka T. Raeed J. M. Wei F.-Y. Shiraki N. Akagi T. (2014). High Oxygen Condition Facilitates the Differentiation of Mouse and Human Pluripotent Stem Cells into Pancreatic Progenitors and Insulin-Producing Cells. J. Biol. Chem. 289, 9623–9638. 10.1074/jbc.m113.524363 24554704 PMC3975012

[B57] Hale M. A. Swift G. H. Hoang C. Q. Deering T. G. Masui T. Lee Y.-K. (2014). The Nuclear Hormone Receptor Family Member NR5A2 Controls Aspects of Multipotent Progenitor Cell Formation and Acinar Differentiation during Pancreatic Organogenesis. Development 141, 3123–3133. 10.1242/dev.109405 25063451 PMC4197540

[B58] Hang Y. Stein R. (2011). MafA and MafB Activity in Pancreatic β Cells. Trends Endocrinol. Metab. 22, 364–373. 10.1016/j.tem.2011.05.003 21719305 PMC3189696

[B59] Harvey A. J. (2007). The Role of Oxygen in Ruminant Preimplantation Embryo Development and Metabolism. Anim. Reprod. Sci. 98, 113–128. 10.1016/j.anireprosci.2006.10.008 17158002

[B60] Heinis M. Simon M.-T. Ilc K. Mazure N. M. Pouysségur J. Scharfmann R. (2010). Oxygen Tension Regulates Pancreatic β-Cell Differentiation through Hypoxia-Inducible Factor 1α. Diabetes 59, 662–669. 10.2337/db09-0891 20009089 PMC2828660

[B61] Hellerström C. Swenne I. (1991). Functional Maturation and Proliferation of Fetal Pancreatic Beta-Cells. Diabetes 40 (Suppl. 2), 89–93. 10.2337/diab.40.2.s89 1748274

[B62] Helman A. Cangelosi A. L. Davis J. C. Pham Q. Rothman A. Faust A. L. (2020). A Nutrient-Sensing Transition at Birth Triggers Glucose-Responsive Insulin Secretion. Cel Metab. 31, 1004–1016. e5. 10.1016/j.cmet.2020.04.004 PMC7480404 32375022

[B63] Hogrebe N. J. Augsornworawat P. Maxwell K. G. Velazco-Cruz L. Millman J. R. (2020). Targeting the Cytoskeleton to Direct Pancreatic Differentiation of Human Pluripotent Stem Cells. Nat. Biotechnol. 38, 460–470. 10.1038/s41587-020-0430-6 32094658 PMC7274216

[B64] Hrvatin S. O’Donnell C. W. Deng F. Millman J. R. Pagliuca F. W. Diiorio P. (2014). Differentiated Human Stem Cells Resemble Fetal, Not Adult, β Cells. Proc. Natl. Acad. Sci. U.S.A. 111, 3038–3043. 10.1073/pnas.1400709111 24516164 PMC3939927

[B65] Huang Y. Karuranga S. Malanda B. Williams D. R. R. (2018). Call for Data Contribution to the IDF Diabetes Atlas 9th Edition 2019. Diabetes Res. Clin. Pract. 140, 351–352. 10.1016/j.diabres.2018.05.033 29871760

[B66] Huising M. O. (2020). Paracrine Regulation of Insulin Secretion. Diabetologia 63, 2057–2063. 10.1007/s00125-020-05213-5 32894316 PMC7968070

[B67] Jaafar R. Tran S. Shah A. N. Sun G. Valdearcos M. Marchetti P. (2019). mTORC1 to AMPK Switching Underlies β-cell Metabolic Plasticity during Maturation and Diabetes. J. Clin. Invest. 129, 4124–4137. 10.1172/JCI127021 31265435 PMC6763225

[B68] Jacovetti C. Matkovich S. J. Rodriguez-Trejo A. Guay C. Regazzi R. (2015). Postnatal β-cell Maturation Is Associated with Islet-specific microRNA Changes Induced by Nutrient Shifts at Weaning. Nat. Commun. 6, 8084. 10.1038/ncomms9084 26330140 PMC4569696

[B69] Jansson L. Hellerström C. (1983). Stimulation by Glucose of the Blood Flow to the Pancreatic Islets of the Rat. Diabetologia 25, 45–50. 10.1007/BF00251896 6350083

[B70] Jun Y. Lee J. Choi S. Yang J. H. Sander M. Chung S. (2019). In Vivo-mimicking Microfluidic Perfusion Culture of Pancreatic Islet Spheroids. Sci. Adv. 5, eaax4520. 10.1126/sciadv.aax4520 31807701 PMC6881167

[B71] Kadison A. Kim J. Maldonado T. Crisera C. Prasadan K. Manna P. (2001). Retinoid Signaling Directs Secondary Lineage Selection in Pancreatic Organogenesis. J. Pediatr. Surg. 36, 1150–1156. 10.1053/jpsu.2001.25734 11479845

[B72] Kane M. A. Folias A. E. Pingitore A. Perri M. Obrochta K. M. Krois C. R. (2010). Identification of 9- Cis -retinoic Acid as a Pancreas-specific Autacoid that Attenuates Glucose-Stimulated Insulin Secretion. Proc. Natl. Acad. Sci. U.S.A. 107, 21884–21889. 10.1073/pnas.1008859107 21115832 PMC3003056

[B73] Komatsu H. Cook C. Wang C.-H. Medrano L. Lin H. Kandeel F. (2017). Oxygen Environment and Islet Size Are the Primary Limiting Factors of Isolated Pancreatic Islet Survival. PLoS One 12, e0183780. 10.1371/journal.pone.0183780 28832685 PMC5568442

[B74] Komatsu H. Kang D. Medrano L. Barriga A. Mendez D. Rawson J. (2016). Isolated Human Islets Require Hyperoxia to Maintain Islet Mass, Metabolism, and Function. Biochem. Biophysical Res. Commun. 470, 534–538. 10.1016/j.bbrc.2016.01.110 26801563

[B75] Kong X. Li B. Deng Y. Ma X. (2019a). FXR Mediates Adenylyl Cyclase 8 Expression in Pancreatic β-Cells. J. Diabetes Res. 2019, 8915818. 10.1155/2019/8915818 31485455 PMC6710725

[B76] Kong X. Feng L. Yan D. Li B. Yang Y. Ma X. (2021). FXR-mediated Epigenetic Regulation of GLP-1R Expression Contributes to Enhanced Incretin Effect in Diabetes after RYGB. J. Cel Mol Med. 10.1111/jcmm.16339 PMC10941525 33611845

[B77] Kong X. Tu Y. Li B. Zhang L. Feng L. Wang L. (2019b). Roux-en-Y Gastric Bypass Enhances Insulin Secretion in Type 2 Diabetes via FXR-Mediated TRPA1 Expression. Mol. Metab. 29, 1–11. 10.1016/j.molmet.2019.08.009 31668381 PMC6728758

[B78] Krivova Y. S. Proshchina A. E. Otlyga D. A. Leonova O. G. Saveliev S. V. (2022). Prenatal Development of Sympathetic Innervation of the Human Pancreas. Ann. Anat. 240, 151880. 10.1016/j.aanat.2021.151880 34896557

[B79] Kroon E. Martinson L. A. Kadoya K. Bang A. G. Kelly O. G. Eliazer S. (2008). Pancreatic Endoderm Derived from Human Embryonic Stem Cells Generates Glucose-Responsive Insulin-Secreting Cells *In Vivo* . Nat. Biotechnol. 26, 443–452. 10.1038/nbt1393 18288110

[B80] Lalloyer F. Vandewalle B. Percevault F. Torpier G. Kerr-Conte J. Oosterveer M. (2006). Peroxisome Proliferator-Activated Receptor α Improves Pancreatic Adaptation to Insulin Resistance in Obese Mice and Reduces Lipotoxicity in Human Islets. Diabetes 55, 1605–1613. 10.2337/db06-0016 16731822

[B81] Lees J. G. Cliff T. S. Gammilonghi A. Ryall J. G. Dalton S. Gardner D. K. (2019). Oxygen Regulates Human Pluripotent Stem Cell Metabolic Flux. Stem Cell Int 2019, 8195614. 10.1155/2019/8195614 PMC6545818 31236115

[B82] Lemos J. R. N. Baidal D. A. Ricordi C. Fuenmayor V. Alvarez A. Alejandro R. (2021). Survival after Islet Transplantation in Subjects with Type 1 Diabetes: Twenty-Year Follow-Up. Diabetes Care 44, e67–e68. 10.2337/dc20-2458 33579716 PMC7985423

[B83] Lin E. E. Scott-Solomon E. Kuruvilla R. (2021). Peripheral Innervation in the Regulation of Glucose Homeostasis. Trends Neurosciences 44, 189–202. 10.1016/j.tins.2020.10.015 PMC7904596 33229051

[B168] Lumelsky N. Blondel O. Laeng P. Velasco I. Ravin R. McKay R. (2001). Differentiation of Embryonic Stem Cells to Insulin-Secreting Structures Similar to Pancreatic Islets. Science 292 (5520), 1389–94. 10.1126/science.1058866 11326082

[B84] Lüscher T. F. Creager M. A. Beckman J. A. Cosentino F. (2003). Diabetes and Vascular Disease: Pathophysiology, Clinical Consequences, and Medical Therapy: Part II. Circulation 108, 1655–1661. 10.1161/01.CIR.0000089189.70578.E2 14517152

[B85] Mamidi A. Prawiro C. Seymour P. A. De Lichtenberg K. H. Jackson A. Serup P. (2018). Mechanosignalling via Integrins Directs Fate Decisions of Pancreatic Progenitors. Nature 564, 114–118. 10.1038/s41586-018-0762-2 30487608

[B86] Marcheva B. Ramsey K. M. Buhr E. D. Kobayashi Y. Su H. Ko C. H. (2010). Disruption of the Clock Components CLOCK and BMAL1 Leads to Hypoinsulinaemia and Diabetes. Nature 466, 627–631. 10.1038/nature09253 20562852 PMC2920067

[B87] Martin C. C. Flemming B. P. Wang Y. Oeser J. K. O'Brien R. M. (2008). Foxa2 and MafA Regulate Islet-specific Glucose-6-Phosphatase Catalytic Subunit-Related Protein Gene Expression. J. Mol. Endocrinol. 41, 315–328. 10.1677/jme-08-0062 18753309 PMC2614309

[B88] Matthews K. A. Rhoten W. B. Driscoll H. K. Chertow B. S. (2004). Vitamin A Deficiency Impairs Fetal Islet Development and Causes Subsequent Glucose Intolerance in Adult Rats. J. Nutr. 134, 1958–1963. 10.1093/jn/134.8.1958 15284383

[B89] Matthews L. C. Hanley N. A. (2011). The Stress of Starvation: Glucocorticoid Restraint of Beta Cell Development. Diabetologia 54, 223–226. 10.1007/s00125-010-1963-x 21072627 PMC3017310

[B90] Mühlbauer E. Bazwinsky-Wutschke I. Wolgast S. Labucay K. Peschke E. (2013). Differential and Day-Time Dependent Expression of Nuclear Receptors RORα, RORβ, RORγ and RXRα in the Rodent Pancreas and Islet. Mol. Cell Endocrinol. 365, 129–138. 10.1016/j.mce.2012.10.001 23073388

[B91] Nair G. G. Liu J. S. Russ H. A. Tran S. Saxton M. S. Chen R. (2019). Recapitulating Endocrine Cell Clustering in Culture Promotes Maturation of Human Stem-Cell-Derived β Cells. Nat. Cel Biol 21, 263–274. 10.1038/s41556-018-0271-4 PMC6746427 30710150

[B92] Navarro G. Xu W. Jacobson D. A. Wicksteed B. Allard C. Zhang G. (2016). Extranuclear Actions of the Androgen Receptor Enhance Glucose-Stimulated Insulin Secretion in the Male. Cel Metab. 23, 837–851. 10.1016/j.cmet.2016.03.015 PMC4864089 27133133

[B93] Ni Q. Gu Y. Xie Y. Yin Q. Zhang H. Nie A. (2017). Raptor Regulates Functional Maturation of Murine Beta Cells. Nat. Commun. 8, 15755. 10.1038/ncomms15755 28598424 PMC5472774

[B94] Noh J.-R. Hwang J. H. Kim Y.-H. Kim K.-S. Gang G.-T. Kim S.-W. (2013). The Orphan Nuclear Receptor Small Heterodimer Partner Negatively Regulates Pancreatic Beta Cell Survival and Hyperglycemia in Multiple Low-Dose Streptozotocin-Induced Type 1 Diabetic Mice. Int. J. Biochem. Cel Biol. 45, 1538–1545. 10.1016/j.biocel.2013.05.004 23680671

[B95] Pagliuca F. W. Millman J. R. Gürtler M. Segel M. Van Dervort A. Ryu J. H. (2014). Generation of Functional Human Pancreatic β Cells *In Vitro* . Cell 159, 428–439. 10.1016/j.cell.2014.09.040 25303535 PMC4617632

[B96] Paneni F. Beckman J. A. Creager M. A. Cosentino F. (2013). Diabetes and Vascular Disease: Pathophysiology, Clinical Consequences, and Medical Therapy: Part I. Eur. Heart J. 34, 2436–2443. 10.1093/eurheartj/eht149 23641007 PMC3743069

[B97] Park K.-G. Lee K.-M. Seo H.-Y. Suh J.-H. Kim H.-S. Wang L. (2007). Glucotoxicity in the INS-1 Rat Insulinoma Cell Line Is Mediated by the Orphan Nuclear Receptor Small Heterodimer Partner. Diabetes 56, 431–437. 10.2337/db06-0753 17259388

[B98] Perelis M. Marcheva B. Ramsey K. M. Schipma M. J. Hutchison A. L. Taguchi A. (2015). Pancreatic β Cell Enhancers Regulate Rhythmic Transcription of Genes Controlling Insulin Secretion. Science 350, aac4250. 10.1126/science.aac4250 26542580 PMC4669216

[B99] Pérez R. J. Benoit Y. D. Gudas L. J. (2013). Deletion of Retinoic Acid Receptor β (RARβ) Impairs Pancreatic Endocrine Differentiation. Exp. Cel Res. 319, 2196–2204. 10.1016/j.yexcr.2013.05.032 PMC3821387 23756134

[B100] Perilhou A. Tourrel-Cuzin C. Zhang P. Kharroubi I. Wang H. Fauveau V. (2008). The MODY1 Gene for Hepatocyte Nuclear Factor 4α and a Feedback Loop Control COUP-TFII Expression in Pancreatic Beta Cells. Mol. Cel Biol 28, 4588–4597. 10.1128/mcb.01191-07 PMC2447131 18474611

[B101] Peterson Q. P. Veres A. Chen L. Slama M. Q. Kenty J. H. R. Hassoun S. (2020). A Method for the Generation of Human Stem Cell-Derived Alpha Cells. Nat. Commun. 11, 2241. 10.1038/s41467-020-16049-3 32382023 PMC7205884

[B102] Petrenko V. Gandasi N. R. Sage D. Tengholm A. Barg S. Dibner C. (2020). In Pancreatic Islets from Type 2 Diabetes Patients, the Dampened Circadian Oscillators lead to Reduced Insulin and Glucagon Exocytosis. Proc. Natl. Acad. Sci. U.S.A. 117, 2484–2495. 10.1073/pnas.1916539117 31964806 PMC7007532

[B103] Philippe J. Missotten M. (1990). Dexamethasone Inhibits Insulin Biosynthesis by Destabilizing Insulin Messenger Ribonucleic Acid in Hamster Insulinoma Cells. Endocrinology 127, 1640–1645. 10.1210/endo-127-4-1640 2169394

[B104] Picard F. Wanatabe M. Schoonjans K. Lydon J. O'Malley B. W. Auwerx J. (2002). Progesterone Receptor Knockout Mice Have an Improved Glucose Homeostasis Secondary to β-cell Proliferation. Proc. Natl. Acad. Sci. U.S.A. 99, 15644–15648. 10.1073/pnas.202612199 12438645 PMC137770

[B105] Popescu I. R. Helleboid-Chapman A. Lucas A. Vandewalle B. Dumont J. Bouchaert E. (2010). The Nuclear Receptor FXR Is Expressed in Pancreatic β-cells and Protects Human Islets from Lipotoxicity. FEBS Lett. 584, 2845–2851. 10.1016/j.febslet.2010.04.068 20447400

[B106] Prentice K. J. Saksi J. Robertson L. T. Lee G. Y. Inouye K. E. Eguchi K. (2021). A Hormone Complex of FABP4 and Nucleoside Kinases Regulates Islet Function. Nature 600, 720. 10.1038/s41586-021-04137-3 34880500 PMC8983123

[B107] Pulimeno P. Mannic T. Sage D. Giovannoni L. Salmon P. Lemeille S. (2013). Autonomous and Self-Sustained Circadian Oscillators Displayed in Human Islet Cells. Diabetologia 56, 497–507. 10.1007/s00125-012-2779-7 23242133 PMC3563957

[B108] Qin J. Chen X. Yu-Lee L.-y. Tsai M.-J. Tsai S. Y. (2010). Nuclear Receptor COUP-TFII Controls Pancreatic Islet Tumor Angiogenesis by Regulating Vascular Endothelial Growth Factor/vascular Endothelial Growth Factor Receptor-2 Signaling. Cancer Res. 70, 8812–8821. 10.1158/0008-5472.can-10-0551 20978203 PMC2970665

[B109] Ranta F. Avram D. Berchtold S. Düfer M. Drews G. Lang F. (2006). Dexamethasone Induces Cell Death in Insulin-Secreting Cells, an Effect Reversed by Exendin-4. Diabetes 55, 1380–1390. 10.2337/db05-1220 16644695

[B110] Reich E. Tamary A. Sionov R. V. Melloul D. (2012). Involvement of Thioredoxin-Interacting Protein (TXNIP) in Glucocorticoid-Mediated Beta Cell Death. Diabetologia 55, 1048–1057. 10.1007/s00125-011-2422-z 22246375

[B111] Renga B. Mencarelli A. Vavassori P. Brancaleone V. Fiorucci S. (2010). The Bile Acid Sensor FXR Regulates Insulin Transcription and Secretion. Biochim. Biophys. Acta (Bba) - Mol. Basis Dis. 1802, 363–372. 10.1016/j.bbadis.2010.01.002 20060466

[B112] Rezania A. Bruin J. E. Arora P. Rubin A. Batushansky I. Asadi A. (2014). Reversal of Diabetes with Insulin-Producing Cells Derived *In Vitro* from Human Pluripotent Stem Cells. Nat. Biotechnol. 32, 1121–1133. 10.1038/nbt.3033 25211370

[B113] Rezania A. Riedel M. J. Wideman R. D. Karanu F. Ao Z. Warnock G. L. (2011). Production of Functional Glucagon-Secreting α-Cells from Human Embryonic Stem Cells. Diabetes 60, 239–247. 10.2337/db10-0573 20971966 PMC3012176

[B114] Rickels M. R. Norris A. W. Hull R. L. (2020). A Tale of Two Pancreases: Exocrine Pathology and Endocrine Dysfunction. Diabetologia 63, 2030–2039. 10.1007/s00125-020-05210-8 32894313 PMC7646259

[B115] Robertson R. P. Harmon J. Tran P. O. Poitout V. (2004). Beta-cell Glucose Toxicity, Lipotoxicity, and Chronic Oxidative Stress in Type 2 Diabetes. Diabetes 53 (Suppl. 1), S119–S124. 10.2337/diabetes.53.2007.s119 14749276

[B116] Roche E. Buteau J. Aniento I. Reig J. A. Soria B. Prentki M. (1999). Palmitate and Oleate Induce the Immediate-Early Response Genes C-Fos and Nur-77 in the Pancreatic Beta-Cell Line INS-1. Diabetes 48, 2007–2014. 10.2337/diabetes.48.10.2007 10512366

[B117] Rodesch F. Simon P. Donner C. Jauniaux E. (1992). Oxygen Measurements in Endometrial and Trophoblastic Tissues during Early Pregnancy. Obstet. Gynecol. 80, 283–285. 1635745

[B118] Rogers M. A. M. Kim C. (2020). Congenital Infections as Contributors to the Onset of Diabetes in Children: A Longitudinal Study in the United States, 2001‐2017. Pediatr. Diabetes 21, 456–459. 10.1111/pedi.12957 31820549 PMC10545449

[B119] Rogers M. A. M. Wei M. Y. Kim C. Lee J. M. (2020). Sex Differences in Autoimmune Multimorbidity in Type 1 Diabetes Mellitus and the Risk of Cardiovascular and Renal Disease: A Longitudinal Study in the United States, 2001-2017. J. Women's Health 29, 511–519. 10.1089/jwh.2019.7935 PMC7194310 32320330

[B120] Rorsman P. Arkhammar P. Bokvist K. Hellerström C. Nilsson T. Welsh M. (1989). Failure of Glucose to Elicit a normal Secretory Response in Fetal Pancreatic Beta Cells Results from Glucose Insensitivity of the ATP-Regulated K+ Channels. Proc. Natl. Acad. Sci. U.S.A. 86, 4505–4509. 10.1073/pnas.86.12.4505 2543980 PMC287299

[B121] Rosario W. Singh I. Wautlet A. Patterson C. Flak J. Becker T. C. (2016). The Brain-To-Pancreatic Islet Neuronal Map Reveals Differential Glucose Regulation from Distinct Hypothalamic Regions. Diabetes 65, 2711–2723. 10.2337/db15-0629 27207534 PMC5001176

[B122] Rosen E. D. Kulkarni R. N. Sarraf P. Ozcan U. Okada T. Hsu C.-H. (2003). Targeted Elimination of Peroxisome Proliferator-Activated Receptor γ in β Cells Leads to Abnormalities in Islet Mass without Compromising Glucose Homeostasis. Mol. Cel Biol 23, 7222–7229. 10.1128/mcb.23.20.7222-7229.2003 PMC230305 14517292

[B123] Russ H. A. Parent A. V. Ringler J. J. Hennings T. G. Nair G. G. Shveygert M. (2015). Controlled Induction of Human Pancreatic Progenitors Produces Functional Beta‐like Cells *In Vitro* . EMBO J. 34, 1759–1772. 10.15252/embj.201591058 25908839 PMC4516429

[B124] Russell R. Carnese P. P. Hennings T. G. Walker E. M. Russ H. A. Liu J. S. (2020). Loss of the Transcription Factor MAFB Limits β-cell Derivation from Human PSCs. Nat. Commun. 11, 2742. 10.1038/s41467-020-16550-9 32488111 PMC7265500

[B125] Saini C. Petrenko V. Pulimeno P. Giovannoni L. Berney T. Hebrok M. (2016). A Functional Circadian Clock Is Required for Proper Insulin Secretion by Human Pancreatic Islet Cells. Diabetes Obes. Metab. 18, 355–365. 10.1111/dom.12616 26662378

[B126] Seo H.-Y. Kim Y. D. Lee K.-M. Min A.-K. Kim M.-K. Kim H.-S. (2008). Endoplasmic Reticulum Stress-Induced Activation of Activating Transcription Factor 6 Decreases Insulin Gene Expression via Up-Regulation of Orphan Nuclear Receptor Small Heterodimer Partner. Endocrinology 149, 3832–3841. 10.1210/en.2008-0015 18450959 PMC2488228

[B127] Shah V. N. Grimsmann J. M. Foster N. C. Dost A. Miller K. M. Pavel M. (2020). Undertreatment of Cardiovascular Risk Factors in the Type 1 Diabetes Exchange Clinic Network ( United States ) and the Prospective Diabetes Follow‐up (Germany/Austria) Registries. Diabetes Obes. Metab. 22, 1577–1585. 10.1111/dom.14069 32329127

[B128] Shapiro A. M. J. Lakey J. R. T. Ryan E. A. Korbutt G. S. Toth E. Warnock G. L. (2000). Islet Transplantation in Seven Patients with Type 1 Diabetes Mellitus Using a Glucocorticoid-free Immunosuppressive Regimen. N. Engl. J. Med. 343, 230–238. 10.1056/nejm200007273430401 10911004

[B129] Shi X. Ma D. Li M. Zeng L. Chen J. Yang Y. (2019). Nuclear Receptor TLX Regulates Islet Beta Cell Proliferation via E2F6. Biochem. Biophysical Res. Commun. 513, 560–566. 10.1016/j.bbrc.2019.04.033 30981507

[B130] Shi X. Xiong X. Dai Z. Deng H. Sun L. Hu X. (2015). Nuclear Orphan Receptor TLX Affects Gene Expression, Proliferation and Cell Apoptosis in Beta Cells. Biochem. Biophysical Res. Commun. 468, 387–393. 10.1016/j.bbrc.2015.10.042 26471300

[B131] Smalls B. L. Ritchwood T. D. Bishu K. G. Egede L. E. (2020). Racial/Ethnic Differences in Glycemic Control in Older Adults with Type 2 Diabetes: United States 2003-2014. Int. J. Environ. Res. Public Health 17. 10.3390/ijerph17030950 PMC7036954 32033032

[B132] Sneddon J. B. Borowiak M. Melton D. A. (2012). Self-renewal of Embryonic-Stem-Cell-Derived Progenitors by Organ-Matched Mesenchyme. Nature 491, 765–768. 10.1038/nature11463 23041930 PMC6005657

[B133] Stafford D. Prince V. E. (2002). Retinoic Acid Signaling Is Required for a Critical Early Step in Zebrafish Pancreatic Development. Curr. Biol. 12, 1215–1220. 10.1016/s0960-9822(02)00929-6 12176331

[B134] Stolovich-Rain M. Enk J. Vikesa J. Nielsen F. C. Saada A. Glaser B. (2015). Weaning Triggers a Maturation Step of Pancreatic β Cells. Develop. Cel 32, 535–545. 10.1016/j.devcel.2015.01.002 25662175

[B135] Strowski M. Z. Parmar R. M. Blake A. D. Schaeffer J. M. (2000). Somatostatin Inhibits Insulin and Glucagon Secretion via Two Receptor Subtypes: An *In Vitro* Study of Pancreatic Islets from Somatostatin Receptor 2 Knockout Mice*. Endocrinology 141, 111–117. 10.1210/endo.141.1.7263 10614629

[B136] Suh Y.-H. Kim S.-Y. Lee H.-Y. Jang B. C. Bae J. H. Sohn J.-N. (2004). Overexpression of Short Heterodimer Partner Recovers Impaired Glucose-Stimulated Insulin Secretion of Pancreatic β-cells Overexpressing UCP2. J. Endocrinol. 183, 133–144. 10.1677/joe.1.05675 15525581

[B137] Susini S. Roche E. Prentki M. Schlegel W. (1998). Glucose and Glucoincretin Peptides Synergize to Induce C‐ Fos , C‐ Jun , junB , Zif ‐268, and Nur‐ 77 Gene Expression in Pancreatic β(INS‐1) Cells. FASEB j. 12, 1173–1182. 10.1096/fasebj.12.12.1173 9737720

[B138] Tahbaz M. Yoshihara E. (2021). Immune Protection of Stem Cell-Derived Islet Cell Therapy for Treating Diabetes. Front. Endocrinol. 12, 716625. 10.3389/fendo.2021.716625 PMC8382875 34447354

[B139] Takahashi K. Tanabe K. Ohnuki M. Narita M. Ichisaka T. Tomoda K. (2007). Induction of Pluripotent Stem Cells from Adult Human Fibroblasts by Defined Factors. Cell 131, 861–872. 10.1016/j.cell.2007.11.019 18035408

[B140] Tan X. Lee L. K. Huynh S. Pawaskar M. Rajpathak S. (2020). Sociodemographic Disparities in the Management of Type 2 Diabetes in the United States. Curr. Med. Res. Opin. 36, 967–976. 10.1080/03007995.2020.1756764 32297530

[B141] Taneera J. Mohammed A. K. Dhaiban S. Hamad M. Prasad R. B. Sulaiman N. (2019). RORB and RORC Associate with Human Islet Dysfunction and Inhibit Insulin Secretion in INS-1 Cells. Islets 11, 10–20. 10.1080/19382014.2019.1566684 30762474 PMC6389281

[B142] Tang T. Abbott M. J. Ahmadian M. Lopes A. B. Wang Y. Sul H. S. (2013). Desnutrin/ATGL Activates PPARδ to Promote Mitochondrial Function for Insulin Secretion in Islet β Cells. Cel Metab. 18, 883–895. 10.1016/j.cmet.2013.10.012 PMC3871209 24268737

[B143] Tessem J. S. Moss L. G. Chao L. C. Arlotto M. Lu D. Jensen M. V. (2014). Nkx6.1 Regulates Islet β-cell Proliferation via Nr4a1 and Nr4a3 Nuclear Receptors. Proc. Natl. Acad. Sci. U.S.A. 111, 5242–5247. 10.1073/pnas.1320953111 24706823 PMC3986138

[B144] Thomson J. A. Itskovitz-Eldor J. Shapiro S. S. Waknitz M. A. Swiergiel J. J. Marshall V. S. (1998). Embryonic Stem Cell Lines Derived from Human Blastocysts. Science 282, 1145–1147. 10.1126/science.282.5391.1145 9804556

[B145] Tiano J. P. Delghingaro-Augusto V. Le May C. Liu S. Kaw M. K. Khuder S. S. (2011). Estrogen Receptor Activation Reduces Lipid Synthesis in Pancreatic Islets and Prevents β Cell Failure in Rodent Models of Type 2 Diabetes. J. Clin. Invest. 121, 3331–3342. 10.1172/jci44564 21747171 PMC3148728

[B146] van der Meulen T. Donaldson C. J. Cáceres E. Hunter A. E. Cowing-Zitron C. Pound L. D. (2015). Urocortin3 Mediates Somatostatin-dependent Negative Feedback Control of Insulin Secretion. Nat. Med. 21, 769–776. 10.1038/nm.3872 26076035 PMC4496282

[B147] Varum S. Rodrigues A. S. Moura M. B. Momcilovic O. Easley C. A. Ramalho-Santos J. (2011). Energy Metabolism in Human Pluripotent Stem Cells and Their Differentiated Counterparts. PLoS One 6, e20914. 10.1371/journal.pone.0020914 21698063 PMC3117868

[B148] Velazco-Cruz L. Goedegebuure M. M. Maxwell K. G. Augsornworawat P. Hogrebe N. J. Millman J. R. (2020a). SIX2 Regulates Human β Cell Differentiation from Stem Cells and Functional Maturation *In Vitro* . Cel Rep. 31, 107687. 10.1016/j.celrep.2020.107687 PMC7304247 32460030

[B149] Velazco-Cruz L. Goedegebuure M. M. Millman J. R. (2020b). Advances toward Engineering Functionally Mature Human Pluripotent Stem Cell-Derived β Cells. Front. Bioeng. Biotechnol. 8, 786. 10.3389/fbioe.2020.00786 32733873 PMC7363766

[B150] Veres A. Faust A. L. Bushnell H. L. Engquist E. N. Kenty J. H.-R. Harb G. (2019). Charting Cellular Identity during Human *In Vitro* β-cell Differentiation. Nature 569, 368–373. 10.1038/s41586-019-1168-5 31068696 PMC6903417

[B151] Vergari E. Knudsen J. G. Ramracheya R. Salehi A. Zhang Q. Adam J. (2019). Insulin Inhibits Glucagon Release by SGLT2-Induced Stimulation of Somatostatin Secretion. Nat. Commun. 10, 139. 10.1038/s41467-018-08193-8 30635569 PMC6329806

[B152] Vieira E. Marroquí L. Batista T. M. Caballero-Garrido E. Carneiro E. M. Boschero A. C. (2012). The Clock GeneRev-Erbα Regulates Pancreatic β-Cell Function: Modulation by Leptin and High-Fat Diet. Endocrinology 153, 592–601. 10.1210/en.2011-1595 22166979

[B153] Wei Z. Yoshihara E. He N. Hah N. Fan W. Pinto A. F. M. (2018). Vitamin D Switches BAF Complexes to Protect β Cells. Cell 173, 1135–1149. 10.1016/j.cell.2018.04.013 29754817 PMC5987229

[B154] Wong W. P. S. Tiano J. P. Liu S. Hewitt S. C. Le May C. Dalle S. (2010). Extranuclear Estrogen Receptor-α Stimulates NeuroD1 Binding to the Insulin Promoter and Favors Insulin Synthesis. Proc. Natl. Acad. Sci. U.S.A. 107, 13057–13062. 10.1073/pnas.0914501107 20616010 PMC2919966

[B155] Wortham M. Sander M. (2021). Transcriptional Mechanisms of Pancreatic Beta-Cell Maturation and Functional Adaptation. Trends Endocrinol. Metab. 10.1016/j.tem.2021.04.011 PMC8259463 34030925

[B156] Xie R. Everett L. J. Lim H.-W. Patel N. A. Schug J. Kroon E. (2013). Dynamic Chromatin Remodeling Mediated by Polycomb Proteins Orchestrates Pancreatic Differentiation of Human Embryonic Stem Cells. Cell Stem Cell 12, 224–237. 10.1016/j.stem.2012.11.023 23318056 PMC3619036

[B157] Yamagata K. Furuta H. Oda N. Kaisaki P. J. Menzel S. Cox N. J. (1996). Mutations in the Hepatocyte Nuclear Factor-4α Gene in Maturity-Onset Diabetes of the Young (MODY1). Nature 384, 458–460. 10.1038/384458a0 8945471

[B158] Yoshihara E. (2020). TXNIP/TBP-2: A Master Regulator for Glucose Homeostasis. Antioxidants (Basel) 9. 10.3390/antiox9080765 PMC7464905 32824669

[B159] Yoshihara E. Chen Z. Matsuo Y. Masutani H. Yodoi J. (2010a). Thiol Redox Transitions by Thioredoxin and Thioredoxin-Binding Protein-2 in Cell Signaling. Methods Enzymol. 474, 67–82. 10.1016/s0076-6879(10)74005-2 20609905

[B160] Yoshihara E. Fujimoto S. Inagaki N. Okawa K. Masaki S. Yodoi J. (2010b). Disruption of TBP-2 Ameliorates Insulin Sensitivity and Secretion without Affecting Obesity. Nat. Commun. 1, 127. 10.1038/ncomms1127 21119640 PMC3060604

[B161] Yoshihara E. Masaki S. Matsuo Y. Chen Z. Tian H. Yodoi J. (2014). Thioredoxin/Txnip: Redoxisome, as a Redox Switch for the Pathogenesis of Diseases. Front. Immunol. 4, 514. 10.3389/fimmu.2013.00514 24409188 PMC3885921

[B162] Yoshihara E. O’Connor C. Gasser E. Wei Z. Oh T. G. Tseng T. W. (2020). Immune-evasive Human Islet-like Organoids Ameliorate Diabetes. Nature 586, 606–611. 10.1038/s41586-020-2631-z 32814902 PMC7872080

[B163] Yoshihara E. Wei Z. Lin C. S. Fang S. Ahmadian M. Kida Y. (2016). ERRγ Is Required for the Metabolic Maturation of Therapeutically Functional Glucose-Responsive β Cells. Cel Metab. 23, 622–634. 10.1016/j.cmet.2016.03.005 PMC4832237 27076077

[B164] Yu C. Cui S. Zong C. Gao W. Xu T. Gao P. (2015). The Orphan Nuclear Receptor NR4A1 Protects Pancreatic β-Cells from Endoplasmic Reticulum (ER) Stress-Mediated Apoptosis. J. Biol. Chem. 290, 20687–20699. 10.1074/jbc.m115.654863 26157144 PMC4543630

[B165] Zhang T. Li X.-H. Zhang D.-B. Liu X.-Y. Zhao F. Lin X.-W. (2017). Repression of COUP-TFI Improves Bone Marrow-Derived Mesenchymal Stem Cell Differentiation into Insulin-Producing Cells. Mol. Ther. - Nucleic Acids 8, 220–231. 10.1016/j.omtn.2017.06.016 28918023 PMC5504083

[B166] Zhou Y.-T. Shimabukuro M. Wang M.-Y. Lee Y. Higa M. Milburn J. L. (1998). Role of Peroxisome Proliferator-Activated Receptor α in Disease of Pancreatic β Cells. Proc. Natl. Acad. Sci. U.S.A. 95, 8898–8903. 10.1073/pnas.95.15.8898 9671776 PMC21174

[B167] Zitzer H. Wente W. Brenner M. B. Sewing S. Buschard K. Gromada J. (2006). Sterol Regulatory Element-Binding Protein 1 Mediates Liver X Receptor-β-Induced Increases in Insulin Secretion and Insulin Messenger Ribonucleic Acid Levels. Endocrinology 147, 3898–3905. 10.1210/en.2005-1483 16644917

