# Supplementary material for: Adapting Physiology in Functional Human Islet Organogenesis
Source: Front Cell Dev Biol. 2022 Apr 26;10:854604. doi: 10.3389/fcell.2022.854604 (PMC9086403; doi:10.3389/fcell.2022.854604)
Supplement: Supplementary file 1 [file DataSheet1.PDF]

Table 1. Physiological role of nuclear receptors in functional β cells.

**Common Name**	**Symbol**	**Abbreviation**	**Ligands**	**Function**	**Human islets (FPKM)**	**Mouse islets (FPKM)**	**Reference**
Thyroid hormone receptor-α	*NR1A1*	*TRα*	*Thyroid hormone*	*Upregulation MAFA, GCK gene expression. Enhances GSIS, insulin production.*	*>10*	*>20*	[118]
Thyroid hormone receptor-β	*NR1A1*	*TRβ*		*Upregulation MAFA, GCK gene expression. Enhances GSIS, insulin production.*	*>1*	*>2*	[118]
Retinoic acid receptor-α	*NR1B1*	*RARα*	*Vitamin A, Retinoic acids*	*Endocrine differentiation. Regulation of β cell mass*	*>5*	*>5*	[126-128]
Retinoic acid receptor-β	*NR1B2*	*RARβ*		*Endocrine differentiation*	*>0.5*	*>0.4*	[128-130]
Retinoic acid receptor-γ	*NR1B3*	*RARγ*		*Endocrine differentiation*	*>1*	*>10*	[128,130]
Peroxisome proliferator-activated receptor-α	*NR1C1*	*PPARα*	*Fatty acids, prostaglandins*	*Enhances fatty acids oxidation and insulin secretion.*	*>3*	*>0.3*	[131,132]
Peroxisome proliferator-activated receptor- δ	*NR1C2*	*PPARδ*		*Enhances fatty acids oxidation and insulin secretion.*	*>6*	*>12*	[133]
Peroxisome proliferator-activated receptor-γ	*NR1C3*	*PPARγ*		*Enhances fatty acids oxidation and insulin secretion.*	*>0.3*	*>5*	[134,135]
Rev-ErbAα	*NR1D1*	*Rev-ErbAα*	*Heme*	*Circadian oscillation.*	*>14*	*>30*	[136,137]
Rev-ErbAα	*NR1D2*	*Rev-ErbAβ*		*Circadian oscillation.*	*>20*	*>30*	[136,137]
RAR-related orphan receptor-α	*NR1F1*	*RORα*	*Cholesterol, retinoic acids*	*Suppress GSIS. Circadian oscillation.*	*>3*	*>1*	[138]
RAR-related orphan receptor-β	*NR1F2*	*RORβ*		*Suppress GSIS. Circadian oscillation.*	*>1*	*>0.01*	[138,139]
RAR-related orphan receptor-γ	*NR1F3*	*RORγ*		*Suppress GSIS. Circadian oscillation.*	*>7*	*>10*	[138,139]
Liver X receptor-β	*NR1H2*	*LXRβ*	*Cholesterol*	*Enhance glycerol/fatty acids cycling.*	*>13*	*>30*	[100-102]

Liver X receptor-α	*NR1H3*	*LXRα*		*Enhance glycerol/fatty acids cycling.*	*>2*	*>5*	[100]
Farnesoid X receptor-α	*NR1H4*	*FXRα*	*Bile acids*	*Upregulation of INS, GLP1R gene expression.*	*>1*	*>9*	[105-108,140]
Farnesoid X receptor-β	*NR1H5*	*FXRβ*		-	-	*>0.01*	-
Vitamin D receptor	*NR1I1*	*VDR*	*Vitamin D*	*Suppress inflammation and prevent cytokine induced β cell dedifferentiation. Modulation of BAF complex.*	*>3*	*>40*	[104]
Pregnane X receptor	*NR1I2*	*PXR*	*xenobiotics*	*Unclear.*	*>0.01*	*>0.05*	-
Constitutive androstane receptor	*NR1I3*	*CAR*	*androstane*	*Unclear.*	*>0.1*	*>0.03*	-
Hepatocyte nuclear factor-4-α	*NR2A1*	*HNF4α*	*Fatty acids*	*Responsible gene for MODY1.*	*>3*	*>10*	[125]
Hepatocyte nuclear factor-4-γ	*NR2A2*	*HNF4γ*		*Pancreatic differentiation*	*>0.2*	*>2*	[141]
Retinoid X receptor-α	*NR2B1*	*RXRα*	*Retinoic acids*	*Pancreatic differentiation.*<br><br>*Attenuate GSIS.*	*>10*	*>12*	[142,143]
Retinoid X receptor-β	*NR2B2*	*RXRβ*		*Pancreatic differentiation.*<br><br>*Attenuate GSIS.*	*>18*	*>18*	[142,143]
Retinoid X receptor-γ	*NR2B3*	*RXRγ*		*Pancreatic differentiation.*<br><br>*Attenuate GSIS.*	*>5*	*>0.1*	[142,143]
Testicular receptor 2	*NR2C1*	*TR2*	-	*Unclear.*	*>3*	*>3*	-
Testicular receptor 4	*NR2C2*	*TR4*		*Unclear.*	*>6*	*>6*	-
Homologue of the Drosophila tailless gene	*NR2E1*	*TLX*	-	*Enhance β cell proliferation.*	*>0.01*	-	[144,145]
Photoreceptor cell-specific nuclear receptor	*NR2E3*	*PNR*	-	*Unclear.*	*>0.01*	*>0.01*	-
Chicken ovalbumin upstream promoter-α	*NR2F1*	*COUP-TFα*	-	*Negatively regulate mouse INS2 gene.*	*>0.5*	*>0.3*	[146-150]

Chicken ovalbumin upstream promoter-β	NR2F2	COUP-TFβ		Positively regulate β cell proliferation. Islet tumorigenesis.	>6	>1	[147,148]
Chicken ovalbumin upstream promoter-γ	NR2F6	COUP-TFγ		Unclear.	>25	>23	-
Estrogen receptor-α	NR3A1	ERα	Estrogens	Regulate insulin synthesis. Suppress Lipid synthesis.	>1	>0.03	[151-153]
Estrogen receptor-β	NR3A2	ERβ		Regulate insulin synthesis. Suppress Lipid synthesis.	>0.01	>0.02	[151-153]
Estrogen-related receptor-α	NR3B1	ERRα		Unclear/Possibly Upregulate mitochondrial gene expression and enhances oxidative metabolism.	>23	>10	-
Estrogen-related receptor-β	NR3B2	ERRβ		Unclear.	>0.1	>0.1	-
Estrogen-related receptor-γ	NR3B3	ERRγ		Upregulate mitochondrial gene expression and enhances oxidative metabolism and postnatal maturation.	>2	>2	[43,47]
Glucocorticoid receptor	NR3C1	GR	Cortisol	Promote pancreatic differentiation. Induces apoptosis.	>14	>17	[112-117,154]
Mineralocorticoid receptor	NR3C2	MR	Aldosterone	Enhance insulin secretion through α cell GLP1 secretion.	>5	>2.5	[155]
Progesterone receptor	NR3C3	PR	Progesterone	Negatively regulate β cell proliferation.	>2	>0.05	[156]
Androgen receptor	NR3C4	AR	Testosterone	Enhances GSIS.	>0.2	>0.1	[103]
Nerve Growth factor IB	NR4A1	NGFIB	-	Positively and negatively regulate β cell proliferation.	>2	>15	[94,98,99]
Nuclear receptor related 1	NR4A2	NURR1	-	Positively regulate β cell proliferation.	>1	>1	[97]
Neuron-derived orphan receptor 1	NR4A3	NOR1	-	Positively and negatively regulate β cell proliferation.	>0.5	>0.05	[94,97,99]
Steroidogenic factor 1	NR5A1	SF-1	Phosphatidylinositols	Unclear.	-	-	-
Liver receptor homolog-1	NR5A2	LRH-1		Pancreas organogenesis. Protect from stress-induced β cell apoptosis.	>2	>0.4	[157-159]
Germ cell nuclear factor	NR6A1	GCNF	-	Unclear.	>0.2	>2.5	-

Dosage-sensitive sex reversal, adrenal hypoplasia critical region, on chromosome X, gene 1	*NR0B1*	*DAX1*	-	*Unclear.*	*>17*	-	-
Small heterodimer partner	*NR0B2*	*SHP*	-	*Negatively regulate β cell survival.*	*>7*	*>0.01*	[160-163]
